# Supplementary material for: Improving outcomes for primary school children at risk of cerebral visual impairment (the CVI project): protocol of a feasibility study for a cluster-randomised controlled trial and health economic evaluation
Source: BMJ Open. 2021 May 5;11(5):e044830. doi: 10.1136/bmjopen-2020-044830 (PMC8103402; doi:10.1136/bmjopen-2020-044830)
Supplement: Supplementary data [file bmjopen-2020-044830supp001.pdf]

CVI Project Parent/Guardian Resource Use Questionnaire

Thank you very much for taking the time to complete this CVI Project parent/guardian resource use questionnaire.

Your answers will be treated in the strictest confidence and will not be shared with anyone outside of the research team. We appreciate you taking part.

We are interested in finding out which school, health and social care services your child has had access to in the last 12 months

1. Please indicate below how many times (if at all) your child has used, or been involved with, any of the services listed below in the last 12 months.
   If your child has not been in contact with a particular service, please enter '0' rather than leave it blank.

Service	Total number of contacts
Hospital outpatient visit (**vision related**, for example, ophthalmologist, orthoptist, hospital optician)	
High street optician	
General Practitioner (GP) (**vision related**)	
QTVI (Qualified Teacher of the Visually Impaired)	

Service	Total number of contacts
Hospital outpatient visit (**not vision related**)	
General Practitioner (GP) (**not vision related**)	
Paediatrician	
Health visitor	
Accident and Emergency (A&E) Visit	
Social Worker	
Hospital stay	
For hospital stay, please record the total number of nights your child has spent in hospital over the last 12 months	Number of nights:
Speech and language therapist	
Hearing specialist	
Occupational therapist	
Physiotherapist	
Child and Adolescent Mental Health Service (CAMHS)	
Educational psychologist	
Dentist	
Police	

Williams C, *et al. BMJ Open* 2021; 11:e044830. doi: 10.1136/bmjopen-2020-044830

If you run out of space, please use the following page to provide additional information.

2. Has your child used any other health and social care services that are not listed in the table above? If so please let us know:

   Other service 1: ____

   No. of contacts: __________

   Other service 2: ____

   No. of contacts: __________

3. In the <u>last 12 months</u>, have you **paid out of your own pocket** (not provided free) for any **<u>services</u>** related to those in the tables or listed above (e.g. private outpatient visit, counselling, homeopathy, educational psychologist)?

   Service 1: ____

   Total spend: __________

   Service 2: ____

   Total spend: __________

4. In the <u>last 12 months</u>, have you **paid out of your own pocket** (not provided free) for any **<u>equipment</u>** related to those in the table or listed above (e.g. additional costs for glasses)?

   Item 1: ____

   Total spend: __________

   Item 2: ____

   Total spend: __________

5. In the <u>last 12 months</u>, did you have to take time off work or your usual daily activities due to your child being off school? (For example, time off due to child's illness, behavioural problems, attending appointments etc.)

Person	NO	YES	If YES, how many days
**Yourself**			
**Partner**			
**Other please say who:**			

Thank you very much for your time.
