# Supplementary material for: Improving outcomes for primary school children at risk of cerebral visual impairment (the CVI project): protocol of a feasibility study for a cluster-randomised controlled trial and health economic evaluation
Source: BMJ Open. 2021 May 5;11(5):e044830. doi: 10.1136/bmjopen-2020-044830 (PMC8103402; doi:10.1136/bmjopen-2020-044830)
Supplement: Supplementary data [file bmjopen-2020-044830supp002.pdf]

Schedule of enrolment, interventions, and assessments

	STUDY PERIOD					
	Enrolment	Allocation	Intervention period		Follow-up	Endpoint
TIMEPOINT (weeks)	*-1 week*	0	*1*	*20*	*21-25*	*26*
**ENROLMENT:**						
**Eligibility screen**	X					
**Informed school consent**	X					
**Randomisation**		X				
**INTERVENTIONS:**						
The CVI Project Intervention			◆———	———◆		
Control standard care			◆———	———◆		
**ASSESSMENTS:**						
Child PedsQL™ generic		X			X	
Child PedsQL™ Cognitive functioning		X			X	
School questionnaire		X			X	
Teacher self-efficacy		X			X	
Teacher Bartimeus CVI		X			X	
Teacher SDQ		X			X	
Teacher PedsQL™ cognitive functioning		X			X	
Teacher Dutton 5 Qs		X			X	
Parent SDQ		X			X	
Parent PedsQL™ Family Impact		X			X	
Parent health costs Qs		X			X	
Parent Dutton 5 Qs		X			X	
Classroom clutter		X			X	
SENCO interviews			X		X	
School staff interviews			X			
Parent interviews			X			
Eye Service staff interviews					X	
Document analysis		X			X	
Functional visual assessments				X		
