# Supplementary material for: Improving outcomes for primary school children at risk of cerebral visual impairment (the CVI project): protocol of a feasibility study for a cluster-randomised controlled trial and health economic evaluation
Source: BMJ Open. 2021 May 5;11(5):e044830. doi: 10.1136/bmjopen-2020-044830 (PMC8103402; doi:10.1136/bmjopen-2020-044830)
Supplement: Author's manuscript [file bmjopen-2020-044830.draft_revisions.pdf]

BMJ Open is committed to open peer review. As part of this commitment we make the peer review history of every article we publish publicly available.

When an article is published we post the peer reviewers' comments and the authors' responses online. We also post the versions of the paper that were used during peer review. These are the versions that the peer review comments apply to.

The versions of the paper that follow are the versions that were submitted during the peer review process. They are not the versions of record or the final published versions. They should not be cited or distributed as the published version of this manuscript.

BMJ Open is an open access journal and the full, final, typeset and author-corrected version of record of the manuscript is available on our site with no access controls, subscription charges or pay-per-view fees (http://bmjopen.bmj.com).

If you have any questions on BMJ Open's open peer review process please email info.bmjopen@bmj.com

BMJ Open

Improving outcomes for primary school children at risk of cerebral visual impairments (The CVI Project): Protocol of a feasibility study for a cluster-randomised controlled trial and health economic evaluation

Journal:	*BMJ Open*
Manuscript ID	bmjopen-2020-044830
Article Type:	Protocol
Date Submitted by the Author:	15-Sep-2020
Complete List of Authors:	Williams, Cathy; University of Bristol<br>Pease, Anna; University of Bristol,<br>Goodenough, Trudy; University of Bristol, Bristol Medical School<br>Breheny, Katie; University of Bristol, Population Health Sciences<br>Gaunt, Daisy; University of Bristol Faculty of Medicine and Dentistry, Bristol Randomised Trials Collaboration & School of Social and Community Medicine; University of Bristol<br>Sinai, Parisa; University of Bristol<br>Watanabe, Rose; University of Bristol
Keywords:	Paediatric ophthalmology < OPHTHALMOLOGY, Developmental neurology & neurodisability < PAEDIATRICS, EPIDEMIOLOGY

SCHOLARONE™ Manuscripts

I, the Submitting Author has the right to grant and does grant on behalf of all authors of the Work (as defined in the below author licence), an exclusive licence and/or a non-exclusive licence for contributions from authors who are: i) UK Crown employees; ii) where BMJ has agreed a CC-BY licence shall apply, and/or iii) in accordance with the terms applicable for US Federal Government officers or employees acting as part of their official duties; on a worldwide, perpetual, irrevocable, royalty-free basis to BMJ Publishing Group Ltd ("BMJ") its licensees and where the relevant Journal is co-owned by BMJ to the co-owners of the Journal, to publish the Work in this journal and any other BMJ products and to exploit all rights, as set out in our licence.

The Submitting Author accepts and understands that any supply made under these terms is made by BMJ to the Submitting Author unless you are acting as an employee on behalf of your employer or a postgraduate student of an affiliated institution which is paying any applicable article publishing charge ("APC") for Open Access articles. Where the Submitting Author wishes to make the Work available on an Open Access basis (and intends to pay the relevant APC), the terms of reuse of such Open Access shall be governed by a Creative Commons licence – details of these licences and which Creative Commons licence will apply to this Work are set out in our licence referred to above.

Other than as permitted in any relevant BMJ Author's Self Archiving Policies, I confirm this Work has not been accepted for publication elsewhere, is not being considered for publication elsewhere and does not duplicate material already published. I confirm all authors consent to publication of this Work and authorise the granting of this licence.

For peer review only

Improving outcomes for primary school children at risk of cerebral visual impairments (The CVI Project): Protocol of a feasibility study for a cluster-randomised controlled trial and health economic evaluation

C Williams [1]; A Pease[1]; T Goodenough [1]; K Breheny[3]; D Gaunt[2]; P Sinai[1] and R Watanabe[1]

[1] Centre for Academic Child Health, Bristol Medical School, University of Bristol, 1-5 Whiteladies Road, BS8 1NU
[2] Bristol Randomised Trials Collaboration, Population Health Sciences, Bristol Medical School, University of Bristol
[3] Population Health Sciences, Bristol Medical School, University of Bristol

Corresponding Author: C Williams, 1-5 Whiteladies Road, Bristol BS8 1NU
Cathy.Williams@bristol.ac.uk

C Williams (orcid.org/0000-0002-9133-2021)
A Pease (orcid.org/0000-0002-3472-1047
T Goodenough (orcid.org/0000-0003-2824-5854)
R Watanabe
P Sinai (orcid.org/0000-0002-8754-2317)
K Breheny(orcid.org/0000-0001-6886-4049.

1-5 Whiteladies Road, Clifton, Bristol, UK, BS8 1NU

D Gaunt

Canynge Hall, 39 Whatley Road, Bristol, UK, BS8 2PS

Word count:3993

For peer review only - http://bmjopen.bmj.com/site/about/guidelines.xhtml

ABSTRACT

Introduction

This paper describes the protocol of a feasibility study for a UK-based cluster randomised controlled trial of a multi-level intervention that aims to improve outcomes for children with cerebral visual impairment (CVI). The effectiveness of this intervention would need to be evaluated in a cluster randomised controlled trial (cRCT) with an economic evaluation. This study is designed to investigate the feasibility of such a trial.

Methods and Analysis

The feasibility study will include all methods anticipated for a future trial and a detailed process evaluation (PE). Primary school children in years 3-5 in eight schools will be recruited. Schools will be randomised to receive the intervention or carry on with usual practice. The intervention will comprise an information pack for schools and access to a local eye clinic (who are prepared to assess them for CVI), for up to 5% of participating children. Outcome assessments will be carried out at baseline and after a 4 to 5 months intervention period. Assessments will include children's self-reported quality of life (QoL) and their learning ability and behaviour as reported by teachers, and family functioning reported by parents. Cost data will include service use, family expenditure on additional support (e.g. cost of private appointments, time spent arranging hospital visits) and school spending and resource used in helping children with special educational needs or disability (SEND). The feasibility of the recruitment and data collection methods will be assessed along with indications of potential intervention effects. The PE will collect data relating to the feasibility and acceptability of the intervention and trial processes. The protocol for this will be reported separately.

Ethics and Dissemination

The results will inform the design of a cRCT to assess the effectiveness and cost-effectiveness of the intervention, if the data suggest this would be feasible, with or without modifications.

Keywords: Cerebral Visual Impairment, Complex intervention, feasibility trial, process evaluation, child, Primary schools.

For peer review only

Strengths and Limitations of this study

This is the first study of a complex intervention that aims to improve outcomes for children with cerebral visual impairment (CVI)

Input from clinicians, parents and teachers has contributed to the design of the intervention and the trial

The data collected will be relevant to policy makers in both education and health

A detailed, mixed methods Process Evaluation (PE) is embedded to inform further development of the intervention and trial methods

The study team will manage and deliver both the feasibility study and the PE, therefore analyses of the PE data will be data-driven to reduce bias

For peer review only

For peer review only - http://bmjopen.bmj.com/site/about/guidelines.xhtml

BACKGROUND

Damage or poor function in the brain areas related to vision leads to brain-related visual impairment, also known as cerebral visual impairment or impairments (CVI) (1). There is no recognised consensus of how CVI should be defined, but a recent review suggested CVI was a verifiable visual impairment not attributable to ocular or optic nerve problems (2). CVI in children can manifest in many ways; for example, being unable to find things in a cluttered page; bumping into people; being unable to copy from the class whiteboard to their own workbooks or difficulties controlling their eye position effectively to keep focused on a task. In a primary school aged child, these difficulties may be misinterpreted as lack of understanding or as clumsiness or inattention, or as social and communication difficulties (3). Children with CVI may not be identified as they may have good visual acuity (4) so are not detected in visual screening programmes recommended for Reception year (age 4-5) children. (5)

The causes and clinical antecedents of CVI are many, including genetic or chromosomal disorders, (6, 7) and perinatal illness or prematurity(8). The impact of CVI will depend on factors within each individual child, their family and their immediate and more distant surroundings. Children with developmental problems and disabilities, who tend to have higher rates of CVI, also have increased risks of additional mental health problems such as anxiety and depression (9). In research by our group, 13-year olds in a UK birth cohort with parent-reported behaviour suggesting CVI achieved less well than expected in their school reading tests at age 13 and were an average of 3-4 months behind their peers, after adjustment for intelligence, socioeconomic status and ocular problems(10).

There are descriptive reports of simple strategies that are helpful for children with CVI, including de-cluttering presentations, using a window to isolate part of the page or using plain backgrounds (11). Also, typically developing children were reported to be more engaged (on task) and retain more information in an uncluttered, base-walled classroom than in a highly decorated classroom (12). However there are no recommended strategies for teachers to use to help support children with vision problems and if these have been diagnosed, the child is usually referred to a specialist teacher for the visually impaired, and they will advise the school.

We have recently completed a study (submitted for publication) with 11 mainstream Primary schools in England and our results suggest that children with CVI-related vision problems are more prevalent than has been appreciated. At least 3.4% of all the children were affected, 80% of who were already recognised by the school as needing extra educational help. Whilst not all of these might be diagnosed with CVI if they presented to a clinic, these data suggest the possibility of improving many children's outcomes by teachers anticipating and supporting their visual needs.

We have developed an intervention that aims to improve outcomes for children with CVI, by sharing information with the teachers and improving links between teachers and their local paediatric ophthalmology clinic. As part of the process described by the Medical Research Council (MRC) for the development of complex interventions,(13) it is now timely to conduct a first evaluation of the potential effectiveness and cost-effectiveness of this intervention and we describe in this paper a feasibility study for a possible future cluster-randomised trial to do this.

For peer review only - http://bmjopen.bmj.com/site/about/guidelines.xhtml

Development of the intervention to improve outcomes for children with CVI

The intervention to be tested was developed in an iterative process, over a two-year period (2017-2019) following guidance on the development of complex interventions (14). The intervention was developed by a range of professionals and some parents, gradually evolving over time. The elements that came together were:

(i) a pragmatic set of tests and assessments that could elicit evidence of specific aspects of CVI, such as poor eye movement control or impaired visuocognitive skills, used with over 200 children in an NHS paediatric ophthalmology clinic run by CW

(ii) discussions and qualitative research with parents, specialist vision teachers (QTVIs) and school staff about support in and out of school, for children with additional needs(15)

(iii) Parent/Patient and Public Involvement meetings (PPI) from groups of parents

(iv) Collation of existing specialist resources published to help parents and teachers manage children with CVI including targeted suggestions for specific problems

(v) Input from international experts in the field including ophthalmologists and paediatricians

The intervention was conceptualised at the outset as "complex" i.e. involving multiple levels, including both educational and health dimensions. The main focus is to inform and empower the school staff, so that they can understand CVI and better support affected children. In parallel, the local NHS paediatric ophthalmology clinic will allow a set number of children at risk of CVI to be assessed, with leaflets detailing advice and support provided to the families and schools of any children then diagnosed with CVI.

The intervention physically comprises: an information pack for schools (a PowerPoint presentation, advice sheets, learning resources for children to raise awareness about vision and sight) and referral letters for up to 5% of children to the local paediatric ophthalmology clinic. For the eye clinic some simple vision tests will be provided and a validated questionnaire to identify possible CVI will be provided plus advice sheets and a report form to be used for the notes to be shared with families and schools.

Patient and Public Involvement

In preliminary work we held meetings with two groups of parents whose children had CVI and/or other additional needs. They commented on the assessment tools (Table 1) and recommended that parents might find some questions off-putting and advised on others that were more acceptable. They chose the Family Impact module above another questionnaire as a tool for capturing the family perspective. Parents suggested that schools would need reimbursing for the teachers' time and that information sheets should emphasize the potential benefits of the intervention to teachers as it may save them time if it enables them to help children to learn better. It would also enable teachers to demonstrate to Ofsted (Office for Standards in Education) inspectors the specific strategies they had used to help struggling learners.

For peer review only - http://bmjopen.bmj.com/site/about/guidelines.xhtml

Aim and objectives

The main aim of this study is to assess the feasibility and acceptability of the intervention and trial methods, to inform the possible development of a future adequately powered cluster-randomised controlled trial.

Our objectives are to collect data on:

1. Recruitment of Primary schools: response rate and yield from flyers, letters, emails and telephone calls; reasons for non-participation, retention once recruited

2. Proportion and predictors of parental non-agreement to data sharing once a school is recruited

3. Outcome assessments: completeness and quality of data from (a) child-report, (b) teacher-report and (c) parent questionnaire

4. Intervention acceptability and feasibility of implementation in the Primary schools and in paediatric ophthalmology clinic

5. Variation in outcome assessment data between and within schools (intracluster correlation) and between the study arms over the course of the study

6. Estimates of cost and resource use connected with supporting children with SEND for schools and for families

7. Number of children suggested by the schools for a referral to the hospital ophthalmology clinic, the proportion that attend and of those, the proportion with any CVIs

8. Unintended consequences of the intervention

For peer review only

Methods and Analysis

Design

The design and timing of the Feasibility study is shown in the flowchart in Figure 1.

Figure 1: The CVI Project Feasibility Trial Flowchart

Setting

The study will involve primary schools in three areas of England, UK: Gloucestershire, Somerset and Hampshire. The study will be led by the study team in Bristol, UK.

Inclusion criteria

For peer review only - http://bmjopen.bmj.com/site/about/guidelines.xhtml

All mainstream Primary schools including academies, local authority maintained and free schools, within one hour of travel time to the local ophthalmology unit, in the three study areas will be

Exclusion criteria

Schools who share a SENCo, where contamination between intervention to control schools might occur unless they are part of a linked group of schools who all participate in the trial (and will be in the same arm).

Enrolment of schools

All schools in the study areas that meet the inclusion criteria will be sent an invitation letter and sample school and parent/carer information sheets. Invitation letters and information sheets will be sent out by post and email to Heads, SENCos and Heads of Governors. The letters and information sheets will explain the study and flag up that schools will be reimbursed for the cost of the teachers' time completing outcome assessments.

Legal basis for conducting the study

The legal basis for the study will be the University of Bristol undertaking a Public task, as described in articles 6.1e and 9.2j of the EU-GDPR. It will not be on the basis of parental consent.

The Head Teacher (HT) will therefore agree to participate, on behalf of the children and staff in their school and will sign a Memorandum of Understanding (MOU) to this effect with clear descriptions of the expectations and obligations of the study team and the participating school. A link to the full Privacy notice online will be provided to all parents and school staff and a summary of the Privacy notice is included in the Parent/ carer information sheet (PIS) and the School information sheet.

Any parent will be able to withdraw their child's data from the study. This can be achieved by contacting the study team using the details in the PIS, or by completing a Non-Agreement to Data Use form that they will all be sent with a PIS, plus a freepost envelope. They will be able to do this at any time up to the end of the study. In addition, if a child does not assent to filling in the child-report questionnaires, they do not have to.

Study set-up and Baseline assessments

An Excel sheet will be completed by the school with name, gender, date of birth (dob), school pupil number (UPN), status regarding free school meals (FSM), siblings at the school and level and reason for extra educational help for all children in Y3-5. Unique study identification numbers (IDs) for each child will be generated. The study team will then provide a Parent Information Sheet (PIS) and a questionnaire for each family, plus self-completion forms with individual study ID numbers, for the children.

Outcome assessments for the future trial were originally selected on the basis of the literature suggesting that unsupported CVI can impair children's learning (10, 16). We have also conducted a

For peer review only

For peer review only - http://bmjopen.bmj.com/site/about/guidelines.xhtml

Core Outcome set (COS) study identifying outcomes relating to CVI in children that were rated as important by families and professionals (in preparation). The outcome assessment questionnaires chosen for this study address many items in the COS: other items will be addressed in interviews with parents and/or in the Process Evaluation. The validated assessment tools to be used are listed in Table 1.

The study team will send or deliver to schools all the materials they need (school and parent information sheets, child questionnaires, parent questionnaires). The parent and child questionnaires will all have a front sheet giving the child's name, class and study ID number, whilst the actual questionnaires will have only the study ID. On completion, the front sheets will be removed and the data returned to the study team with study ID only, by freepost envelope for the parent questionnaires and child-report forms will be collected from the school by a member of the study team.

Teachers will get a link to an online questionnaire generated by a REDCap database and will complete one for each child in their class, plus answer a Self-efficacy questionnaire relating to their own teaching practice.

Table 1. Assessment tools to be used in the Feasibility study

Respondent	Concept being investigated	Tool	Format
Child	Quality of Life	PedsQL Generic Core Scales: *Child -report 8-12y OR* *Child report 5-7y (supported by adult report)*	Paper<br>Which form the child completes is at discretion of their teacher
Teacher	Child learning ability	PEDSQoL Cognitive Scales	Online REDCap form– one per child
	Child behaviour at school	SDQ with impact supplement	Online REDCap form – one per child
	Child behaviour that may indicate CVIs	5-item CVI questionnaire	Online REDCap form– one per child
	Teacher perception of their own teaching	Self-Efficacy	Online REDCap form – one per teacher
Parents	Parental Quality of Life related to family	PEDSQL Family Impact Module	Paper<br>Parents fill in one per child in the study
	Generic and child related costs and resource input	Family expenses questionnaire (designed for the study)	Paper<br>Parents fill in one per child in the study
	Child behaviour that may indicate CVIs	5-item CVI questionnaire	Paper<br>Parents fill in one per child in the study

For peer review only - http://bmjopen.bmj.com/site/about/guidelines.xhtml

Randomisation

After baseline data are collected, a statistician separate from the study will randomise the participating schools, with two stratification variables that might modify the effectiveness of the intervention: (a) 15% or less of children having SEND vs >15% and (b) 3+ classes per year vs 2 or fewer classes per year.

The schools will be informed of their allocation by email and telephone call, all on the same day.

Post-randomisation

Immediately after group allocation, the intervention will be given to schools in the intervention arm. It will be sent by email with paper copies supplied by post or delivered and access to a password-protected link on the study website will be given, for further downloads. The local PIs in the paediatric ophthalmology clinics will be given the CVI test equipment, advice leaflets and a link to a password-protected secure REDCap online form to record CVI-related test data.

Study Intervention components

The individual elements of the intervention are:

School-level

Resources with materials for use in a staff meeting or other staff professional development session, containing:

(i) A short (30 min) presentation with audio commentary by the study PI (CW) giving an overview of CVI and how to help affected children and of the other materials provided, plus a transcript of the presentation.

(ii) Links to online resources about CVI

(iii) A "Toolkit"- Word document detailing simple, inexpensive strategies (a) for universal use or (b) to help individual children who are struggling with learning.

(iv) Some teaching resources (stories, suggested activities for children to do), matched to the curriculum, for teachers to use to help explain topics related to vision and light.

(v) Letters for teachers to give to parents to show their GP, requesting referral to the local paediatric ophthalmology clinic because vision problems are suspected, for up to 5% of participating children. This limit was set to avoid overburdening the GPs and ophthalmology clinic.

It is anticipated that all teaching staff will view the presentation and be made aware of the topic, whilst the SENCo and some of the LSAs would also access the other materials at a time suited to them. Whether this happens as intended will be investigated in the PE.

NHS-level

For peer review only - http://bmjopen.bmj.com/site/about/guidelines.xhtml

The processes involved in the NHS component are summarised in Figure 2.

The study team will recruit a Paediatric Ophthalmologist (PO) in the local hospital as a collaborator, who will agree to oversee the assessment of children from the study who may be referred by their GP in response to a suggestion by the school. Intervention schools will be able to suggest referral of a maximum of 5% of children in years 3-5 for a CVI assessment. Study children who are referred by their GP will be offered an appointment for a CVI assessment and sent an information sheet and a consent form. At the appointment the parent's consent and child's assent will be confirmed. The aim of the CVI assessment is to understand whether the children who are referred do indeed have the target condition or not. This is research data that will be submitted to the study team via a secure REDCap link. The Trusts will be reimbursed for the time taken to collect this. Further management of the child will be in routine clinical care at the orthoptist's discretion and will not be shared with the study team. The orthoptist and paediatric ophthalmologist will have access to the CVI assessment data and will be able to include a printout in the child's notes. The clinicians will be given advice sheets with strategies to help, to give to families following the CVI assessment, if wanted.

.

Control Schools

Schools allocated to the control group will be asked to carry on as normal for the school year.

Process Evaluation (PE)

A detailed investigation of the processes involved in the study will be carried out, involving interviews with staff and parents, observations in schools and analysis of school documents. These data will be used to investigate the assumptions on which the intervention is based - for example that informing teachers about CVI and how to help will actually change their behaviour and that in turn will lead to improved outcomes for the children. The methods for the Process Evaluation will be published in a separate protocol. The primary focus will be in the schools, but interviews and process evaluation data will be collected in the paediatric ophthalmology clinics as well. Briefly, the objectives of the PE are:

1. To assess the implementation of the intervention: fidelity, reach, dose and acceptability, in schools and in the paediatric ophthalmology clinics

2. To investigate the mechanisms of action described by the intervention Logic Model

3. To assess the acceptability of the trial methods including recruitment and data collection, in the schools and the paediatric ophthalmology clinic

4. To explore any unintended consequences resulting from the intervention or trial

Health Economic Evaluation

It is anticipated that a future cluster-RCT will involve a cost-utility analysis (CUA), from a public sector perspective that includes family, education and health service costs. We will collect data from parents on their children's use of NHS primary and secondary care resource use and any additional costs incurred to support their child's health and educational needs. This will include time spent

For peer review only - http://bmjopen.bmj.com/site/about/guidelines.xhtml

pursuing specialist appointments and other administrative tasks as well as costs related to their child's vision.

We will collect data from schools about the resource they allocate to supporting children with SEND, in terms of numbers and hours of Learning Support Assistants (LSAs), costs of any special equipment, and requests for external specialists e.g. Speech and Language therapists. We will collect data from the paediatric ophthalmology clinics about numbers of children who attend the assessment, the staff who would normally assess such children, the average time for appointments and the cost of equipment needed. The costs of delivering the intervention by schools will be explored in the process evaluation. Preliminary work, including the COS development, identified the PedsQL as a potential primary effectiveness outcome for the study. There is extensive literature (17) mapping the PedsQL to the CHU9D to facilitate the calculation of quality adjusted life years (QALYs), therefore it was decided that to reduce questionnaire burden, a preference based questionnaire (e.g. CHU9D) would not be included in the feasibility study. . The possibility of alternative frameworks including broader outcomes, such as cost-consequence analyses (CCA), will also be explored.

Blinding and contamination

It will not be possible to blind school staff as to which arm of the study they are in, or study fieldwork staff. Analysis will be carried out by a separate statistician who is blind to group allocation. The primary outcome for the definitive trial is planned to be the child self-report (see Table 1) which should be less subject to allocation bias than the teacher-report would be. The feasibility of collecting the child-report data is therefore an important outcome from this study. The intervention group schools will be asked not to share the intervention materials however it is recognised that contamination between arms may occur and the PE will investigate this.

Sample Size

As this is a feasibility study, formal power calculations are not appropriate. In previous planning sessions, it was anticipated that a large number of children (3840) would be needed in order to be able to include approximately 20 children in each arm with CVIs, which was arbitrarily set as a pragmatic goal. This assumed a prevalence for CVIs of 1% in mainstream schools and data completion by 75% of participants. Early versions of the Protocol for the feasibility study included these numbers and modelled that they could be recruited from 16 mainstream schools, each contributing 240 children and 1- 2 special schools contributing additional affected children as needed.

However, our recent survey and case-finding study (submitted for publication) suggested a higher prevalence of CVIs (3.4%, largely among SEND children) and recruitment of schools to the survey was harder than expected.

We therefore amended the earlier plans and aim to recruit 1300 children, assuming data returns of 95% and a prevalence of 3.5% of CVIs, to give approximately 21 in each arm. The number of schools and classes will depend on the class sizes in years 3-5 of the participating schools.

Analysis

Statistical analysis of the quantitative data from this study will be carried out by a statistician from the Bristol Randomised Trials Collaborative (BRTC) and a detailed analysis plan will be prepared in

For peer review only - http://bmjopen.bmj.com/site/about/guidelines.xhtml

advance. For between arm comparisons, an Intention-to-Treat approach will be used. Analyses will be mainly descriptive and will include 95% confidence intervals (95% CI) wherever appropriate.

Recruitment

We will report the numbers of flyers and invitation letters sent by post and email, and the yield of enquiries and schools signing up. Once recruited we will report the numbers and proportions of children whose parents refuse to share their data. We will compare these results with those published by other trials involving Primary schools and consider whether any changes in approach would be needed for a full trial.

Outcome assessment data collection

We will report the numbers and proportions of participating children for whom we receive child-report, teacher-report and parent-report outcome data, both at baseline and at follow-up. We will report the proportion of missing data within returned questionnaires.

The results of our earlier survey suggest that 80% of children with CVIs are within the larger group of SEND children. We will plan to assess the primary outcomes in the subgroup of children with SEND in the future trial and carry out secondary analyses including all children. We will therefore assess the variability in outcome data comparing the results for SEND children with those for all children. We will estimate the intracluster correlation coefficient (ICC) for the assessment tools, to inform power calculations for the future trial, together with published ICC data from completed studies.

Health Economic Evaluation

The health economic analysis will also be predominantly descriptive. Item-level missing data and the return of adult and child questionnaires will be explored. As the intervention is predominantly delivered outside of the NHS, data on individual level resource use in this study is mostly reliant upon parent report. The data quality will be explored to inform the refinement of questionnaires and utility of pursuing linked data collection in a future trial. Completion and return of school and NHS eye clinic data will also be examined.

Governance

The study will be managed day-to-day by the study team. Expert advice will be available from the study Advisory groups (one each for parents and professionals). A Trial Steering Committee (TSC), chaired by an independent expert academic, will oversee the conduct of the study. The TSC will discuss the study results and findings with the study team and will advise on the final decision of whether to apply for funding for a full trial, with modifications to the intervention or study methods if indicated.

Ethics and Dissemination

For peer review only - http://bmjopen.bmj.com/site/about/guidelines.xhtml

For peer review only

Ethics and registration

Ethical approval for the protocol of this study (version 2) was obtained from the University of Bristol Faculty of Health Sciences Ethics Committee (FREC Ref: 89144) in August 2019. The study is registered on the International Standard Randomised Controlled Trial Number registry (registration number: ISRCTN13762177). Approval from the Health Research Authority (HRA- Ref 19/HRA/6124) has been obtained.

Dissemination

Results from this study will be submitted to peer-reviewed journals and presented at scientific conferences. Summaries will be provided on the study website and for family support group websites and social media platforms. A meeting for all participants and collaborators will be held to present the findings. If indicated, the results will be used in a funding application for a future trial.

For peer review only

Trial status

Recruitment for this trial began in August 2019 and closed in December 2019. Baseline data were collected in December 2019 and January 2020, then the schools were randomised and informed of their group allocation. Intervention packs were delivered to schools in March 2020. The study was paused in March 2020 because of the corona virus pandemic (covid19). At the time of submission (14 September 2020), we have obtained Faculty Ethical committee (FREC) and Health Research Authority (HRA) permission to restart the study, with minor amendments to the protocol in line with covid19 precautions. Follow-up data will be collected in January 2021. The covid19 amendments will be reported with the results.

Acknowledgements

We are very grateful to Mai Baquedano for her help and innovation with the REDCap database, to Cassandra Wye for the stories and learning resources and to Professor Innes Cuthill for advice and support with measuring visual clutter.

Author contributions

CW designed and led the study, with input from all authors. AP and TG led and designed the Process Evaluation. PS provided governance support and RW managed the databases. KB designed and led the Economic Evaluation and DG provided statistical support. All authors contributed to and approved the final manuscript.

For peer review only - http://bmjopen.bmj.com/site/about/guidelines.xhtml

Funding and Disclaimer

This study presents independent research funded by the National Institute for Health Research (NIHR) Senior Fellowship award SRF_08_005. The views expressed are those of the author(s) and not necessarily those of the NHS, the NIHR or the Department of Health and Social Care.

Competing Interests

None

Legend to Table 1

References for Assessment tools:
PedsQoL Generic Core Scales (18)
PedsQoL Cognitive Scales (19)
PedsQoL Family Impact Module (20)
Strengths and Difficulties Questionnaire (21)
CVI 5 Questions (22)
Teachers Self Efficacy (23)

References

1. Dutton GN. Cognitive vision, its disorders and differential diagnosis in adults and children: knowing where and what things are. Eye (London, England). 2003;17(3):289-304.
2. Sakki HEA, Dale NJ, Sargent J, Perez-Roche T, Bowman R. Is there consensus in defining childhood cerebral visual impairment? A systematic review of terminology and definitions. The British journal of ophthalmology. 2018;102(4):424-32.
3. Swift S, Davidson R, Weems L. Cortical Visual Impairment: Presentation Intervention and Prognosis in Educational Settings. TEACHING Exceptional Children PLus. 2008;4(5):1-14.
4. van Genderen M, Dekker M, Pilon F, Bals I. Diagnosing cerebral visual impairment in children with good visual acuity. Strabismus. 2012;20(2):78-83.
5. Solebo AL, Cumberland PM, Rahi JS. Whole-population vision screening in children aged 4-5 years to detect amblyopia. Lancet. 2015;385(9984):2308-19.
6. Bosch DG, Boonstra FN, de Leeuw N, Pfundt R, Nillesen WM, de Ligt J, et al. Novel genetic causes for cerebral visual impairment. European journal of human genetics : EJHG. 2016;24(5):660-5.
7. Bosch DG, Boonstra FN, Reijnders MR, Pfundt R, Cremers FP, de Vries BB. Chromosomal aberrations in cerebral visual impairment. European journal of paediatric neurology : EJPN : official journal of the European Paediatric Neurology Society. 2014;18(6):677-84.
8. Dutton GN. The spectrum of cerebral visual impairment as a sequel to premature birth: an overview. Documenta ophthalmologica Advances in ophthalmology. 2013;127(1):69-78.

For peer review only - http://bmjopen.bmj.com/site/about/guidelines.xhtml

9. Murphy M, Fonagy P. Chief Medical Officers Report 2012. Chapter 10. Mental health problems in children and young people. In: Health UgDo, editor.: UK Government; 2012.
10. Williams C, Northstone K, Sabates R, Feinstein L, Emond A, Dutton GN. Visual perceptual difficulties and under-achievement at school in a large community-based sample of children. PLoS One. 2011;6(3):e14772.
11. Philip SS, Dutton GN. Identifying and characterising cerebral visual impairment in children: a review. Clinical & experimental optometry. 2014;97(3):196-208.
12. Fisher AV, Godwin KE, Seltman H. Visual environment, attention allocation, and learning in young children: when too much of a good thing may be bad. Psychol Sci. 2014;25(7):1362-70.
13. Craig P, Dieppe P, Macintyre S, Michie S, Nazareth I, Petticrew M, et al. Developing and evaluating complex interventions: the new Medical Research Council guidance. BMJ. 2008;337:a1655.
14. O'Cathain A, Croot L, Duncan E, Rousseau N, Sworn K, Turner KM, et al. Guidance on how to develop complex interventions to improve health and healthcare. BMJ open. 2019;9(8):e029954.
15. Pease A, Goodenough T, Roe J, Rodgers S, Williams C. Perspectives of primary school staff who work with children with additional needs. BJVI. 2020;online first.
16. Molloy CS, Di Battista AM, Anderson VA, Burnett A, Lee KJ, Roberts G, et al. The contribution of visual processing to academic achievement in adolescents born extremely preterm or extremely low birth weight. Child Neuropsychol. 2017;23(3):361-79.
17. Sweeney R, Chen G, Gold L, Mensah F, Wake M. Mapping PedsQL(TM) scores onto CHU9D utility scores: estimation, validation and a comparison of alternative instrument versions. Qual Life Res. 2020;29(3):639-52.
18. Varni JW, Seid M, Kurtin PS. PedsQL 4.0: reliability and validity of the Pediatric Quality of Life Inventory version 4.0 generic core scales in healthy and patient populations. Med Care. 2001;39(8):800-12.
19. Varni JW, Limbers CA, Sorensen LG, Neighbors K, Martz K, Bucuvalas JC, et al. PedsQL Cognitive Functioning Scale in pediatric liver transplant recipients: feasibility, reliability, and validity. Qual Life Res. 2011;20(6):913-21.
20. Varni JW, Sherman SA, Burwinkle TM, Dickinson PE, Dixon P. The PedsQL Family Impact Module: preliminary reliability and validity. Health Qual Life Outcomes. 2004;2:55.
21. Goodman R. The extended version of the Strengths and Difficulties Questionnaire as a guide to child psychiatric caseness and consequent burden. J Child Psychol Psychiatry. 1999;40(5):791-9.
22. Gorrie F, Goodall K, Rush R, Ravenscroft J. Towards population screening for Cerebral Visual Impairment: Validity of the Five Questions and the CVI Questionnaire. PLoS One. 2019;14(3):e0214290.
23. Schwarzer R, Schmitz, GS , Daytner G. Teacher Self-efficacy 1999

For peer review only

For peer review only - http://bmjopen.bmj.com/site/about/guidelines.xhtml

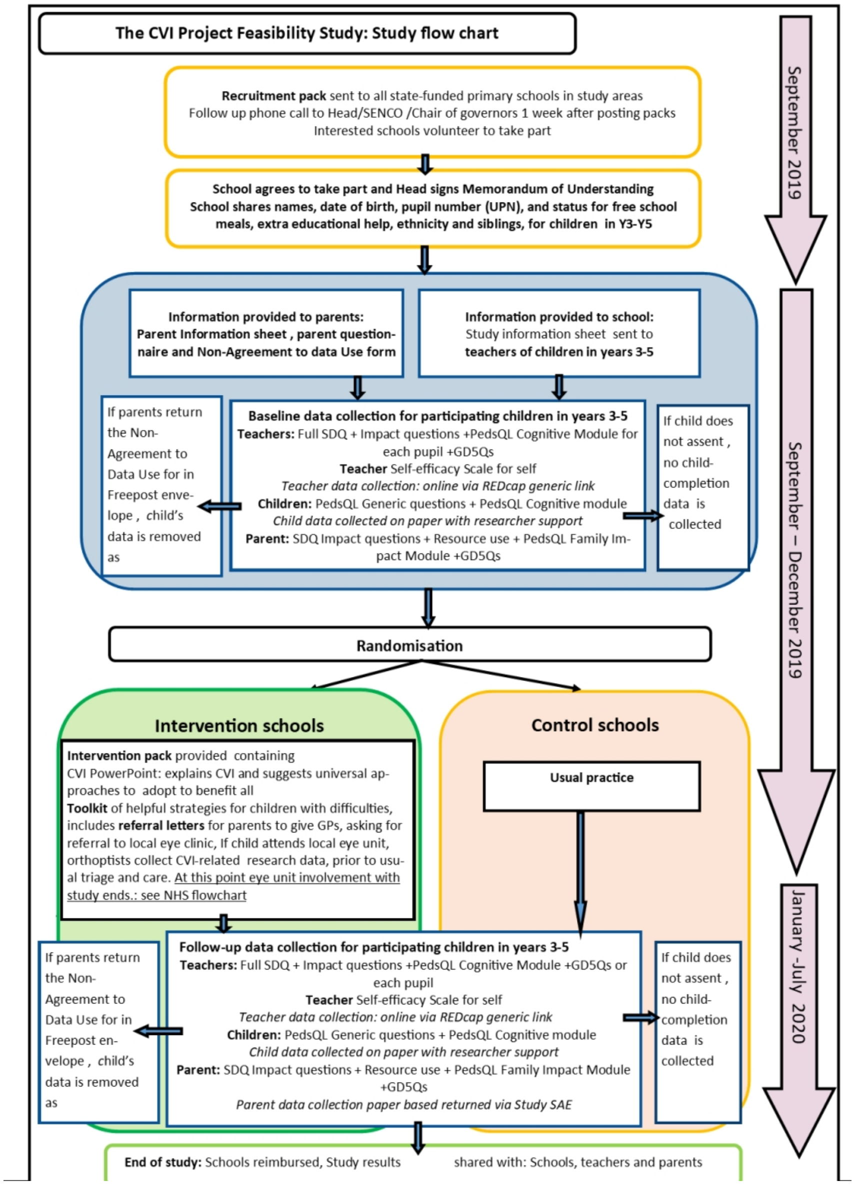
The CVI Project Feasibility Study: Study flow chart
Recruitment pack sent to all state-funded primary schools in study areas
Follow up phone call to Head/SENCO /Chair of governors 1 week after posting packs
Interested schools volunteer to take part
School agrees to take part and Head signs Memorandum of Understanding
School shares names, date of birth, pupil number (UPN), and status for free school meals, extra educational help, ethnicity and siblings, for children in Y3-Y5
Information provided to parents: Parent Information sheet , parent questionnaire and Non-Agreement to data Use form
Information provided to school: Study information sheet sent to teachers of children in years 3-5
If parents return the Non-Agreement to Data Use for in Freepost envelope , child's data is removed as
Baseline data collection for participating children in years 3-5
Teachers: Full SDQ + Impact questions +PedsQL Cognitive Module for each pupil +GD5Qs
Teacher Self-efficacy Scale for self
Teacher data collection: online via REDcap generic link
Children: PedsQL Generic questions + PedsQL Cognitive module
Child data collected on paper with researcher support
Parent: SDQ Impact questions + Resource use + PedsQL Family Impact Module +GD5Qs
If child does not assent , no child-completion data is collected
Randomisation
Intervention schools
Intervention pack provided containing
CVI PowerPoint: explains CVI and suggests universal approaches to adopt to benefit all
Toolkit of helpful strategies for children with difficulties, includes referral letters for parents to give GPs, asking for referral to local eye clinic, If child attends local eye unit, orthoptists collect CVI-related research data, prior to usual triage and care. At this point eye unit involvement with study ends.: see NHS flowchart
Control schools
Usual practice
If parents return the Non-Agreement to Data Use for in Freepost envelope , child's data is removed as
Follow-up data collection for participating children in years 3-5
Teachers: Full SDQ + Impact questions +PedsQL Cognitive Module +GD5Qs or each pupil
Teacher Self-efficacy Scale for self
Teacher data collection: online via REDcap generic link
Children: PedsQL Generic questions + PedsQL Cognitive module
Child data collected on paper with researcher support
Parent: SDQ Impact questions + Resource use + PedsQL Family Impact Module +GD5Qs
Parent data collection paper based returned via Study SAE
If child does not assent , no child-completion data is collected
End of study: Schools reimbursed, Study results shared with: Schools, teachers and parents
September 2019
September – December 2019
January -July 2020

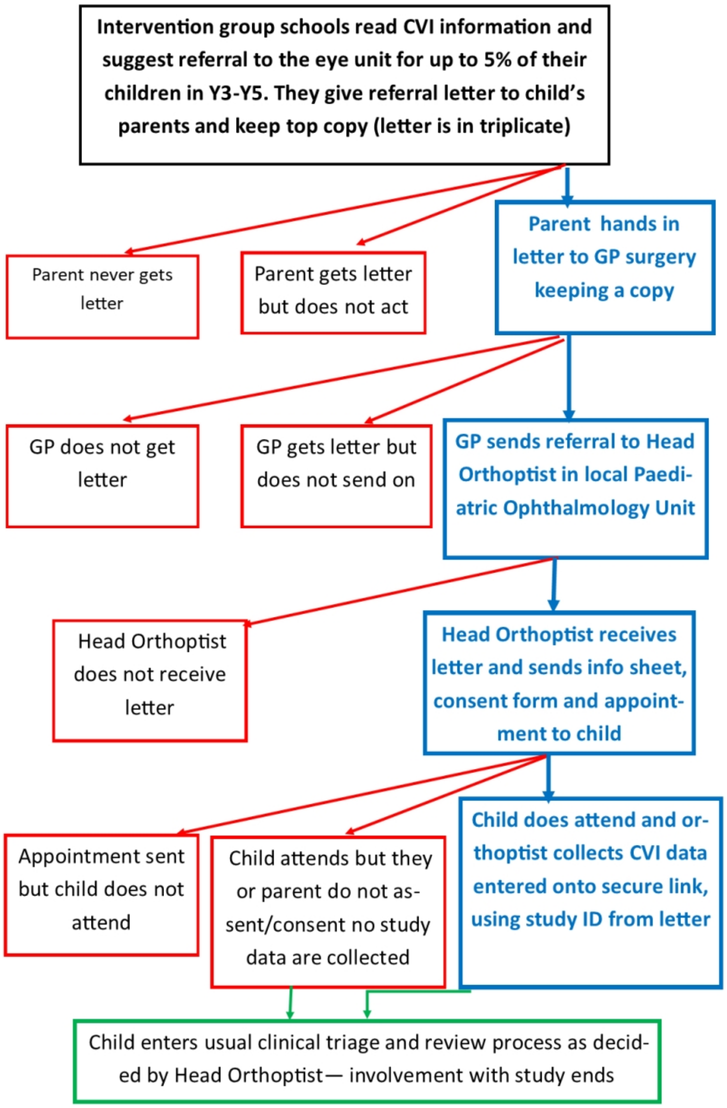
Intervention group schools read CVI information and suggest referral to the eye unit for up to 5% of their children in Y3-Y5. They give referral letter to child's parents and keep top copy (letter is in triplicate)
Parent never gets letter
Parent gets letter but does not act
Parent hands in letter to GP surgery keeping a copy
GP does not get letter
GP gets letter but does not send on
GP sends referral to Head Orthoptist in local Paediatric Ophthalmology Unit
Head Orthoptist does not receive letter
Head Orthoptist receives letter and sends info sheet, consent form and appointment to child
Appointment sent but child does not attend
Child attends but they or parent do not assent/consent no study data are collected
Child does attend and orthoptist collects CVI data entered onto secure link, using study ID from letter
Child enters usual clinical triage and review process as decided by Head Orthoptist— involvement with study ends

STANDARD PROTOCOL ITEMS: RECOMMENDATIONS FOR INTERVENTIONAL TRIALS

SPIRIT 2013 Checklist: Recommended items to address in a clinical trial protocol and related documents*

Section/item	Item No	Description	Addressed on page number
**Administrative information**			
Title	1	Descriptive title identifying the study design, population, interventions, and, if applicable, trial acronym	Title__________
Trial registration	2a	Trial identifier and registry name. If not yet registered, name of intended registry	_p13__________
	2b	All items from the World Health Organization Trial Registration Data Set	___________
Protocol version	3	Date and version identifier	__p13_________
Funding	4	Sources and types of financial, material, and other support	__p14__________
Roles and responsibilities	5a	Names, affiliations, and roles of protocol contributors	__p1 and p13____
	5b	Name and contact information for the trial sponsor	______________
	5c	Role of study sponsor and funders, if any, in study design; collection, management, analysis, and interpretation of data; writing of the report; and the decision to submit the report for publication, including whether they will have ultimate authority over any of these activities	______________
	5d	Composition, roles, and responsibilities of the coordinating centre, steering committee, endpoint adjudication committee, data management team, and other individuals or groups overseeing the trial, if applicable (see Item 21a for data monitoring committee)	______________

For peer review only

**Introduction**			
Background and rationale	6a	Description of research question and justification for undertaking the trial, including summary of relevant studies (published and unpublished) examining benefits and harms for each intervention	_____p4________
	6b	Explanation for choice of comparators	_____p12_________
Objectives	7	Specific objectives or hypotheses	_____p6______
Trial design	8	Description of trial design including type of trial (eg, parallel group, crossover, factorial, single group), allocation ratio, and framework (eg, superiority, equivalence, noninferiority, exploratory)	_____pp6-10__
**Methods: Participants, interventions, and outcomes**			
Study setting	9	Description of study settings (eg, community clinic, academic hospital) and list of countries where data will be collected. Reference to where list of study sites can be obtained	_____p6_______
Eligibility criteria	10	Inclusion and exclusion criteria for participants. If applicable, eligibility criteria for study centres and individuals who will perform the interventions (eg, surgeons, psychotherapists)	______pp6=7______
Interventions	11a	Interventions for each group with sufficient detail to allow replication, including how and when they will be administered	_____p8__________
	11b	Criteria for discontinuing or modifying allocated interventions for a given trial participant (eg, drug dose change in response to harms, participant request, or improving/worsening disease)	______n/a________
	11c	Strategies to improve adherence to intervention protocols, and any procedures for monitoring adherence (eg, drug tablet return, laboratory tests)	_____p10_________
	11d	Relevant concomitant care and interventions that are permitted or prohibited during the trial	_____n/a_________
Outcomes	12	Primary, secondary, and other outcomes, including the specific measurement variable (eg, systolic blood pressure), analysis metric (eg, change from baseline, final value, time to event), method of aggregation (eg, median, proportion), and time point for each outcome. Explanation of the clinical relevance of chosen efficacy and harm outcomes is strongly recommended	___p8_______
Participant timeline	13	Time schedule of enrolment, interventions (including any run-ins and washouts), assessments, and visits for participants. A schematic diagram is highly recommended (see Figure)	see Figure_________

For peer review only - http://bmjopen.bmj.com/site/about/guidelines.xhtml

Sample size	14	Estimated number of participants needed to achieve study objectives and how it was determined, including clinical and statistical assumptions supporting any sample size calculations	_p11___________
Recruitment	15	Strategies for achieving adequate participant enrolment to reach target sample size	_p7___________

Methods: Assignment of interventions (for controlled trials)

Allocation:

Sequence generation	16a	Method of generating the allocation sequence (eg, computer-generated random numbers), and list of any factors for stratification. To reduce predictability of a random sequence, details of any planned restriction (eg, blocking) should be provided in a separate document that is unavailable to those who enrol participants or assign interventions	__p9__________
Allocation concealment mechanism	16b	Mechanism of implementing the allocation sequence (eg, central telephone; sequentially numbered, opaque, sealed envelopes), describing any steps to conceal the sequence until interventions are assigned	_p9____________
Implementation	16c	Who will generate the allocation sequence, who will enrol participants, and who will assign participants to interventions	_p9____________
Blinding (masking)	17a	Who will be blinded after assignment to interventions (eg, trial participants, care providers, outcome assessors, data analysts), and how	_p11___________
	17b	If blinded, circumstances under which unblinding is permissible, and procedure for revealing a participant's allocated intervention during the trial	__n/a__________

Methods: Data collection, management, and analysis

Data collection methods	18a	Plans for assessment and collection of outcome, baseline, and other trial data, including any related processes to promote data quality (eg, duplicate measurements, training of assessors) and a description of study instruments (eg, questionnaires, laboratory tests) along with their reliability and validity, if known. Reference to where data collection forms can be found, if not in the protocol	P8_________

	18b	Plans to promote participant retention and complete follow-up, including list of any outcome data to be collected for participants who discontinue or deviate from intervention protocols	_____p10_______
Data management	19	Plans for data entry, coding, security, and storage, including any related processes to promote data quality (eg, double data entry; range checks for data values). Reference to where details of data management procedures can be found, if not in the protocol	____p8________
Statistical methods	20a	Statistical methods for analysing primary and secondary outcomes. Reference to where other details of the statistical analysis plan can be found, if not in the protocol	_p11-12_______
	20b	Methods for any additional analyses (eg, subgroup and adjusted analyses)	p11-12________
	20c	Definition of analysis population relating to protocol non-adherence (eg, as randomised analysis), and any statistical methods to handle missing data (eg, multiple imputation)	_n/a_________
**Methods: Monitoring**			
Data monitoring	21a	Composition of data monitoring committee (DMC); summary of its role and reporting structure; statement of whether it is independent from the sponsor and competing interests; and reference to where further details about its charter can be found, if not in the protocol. Alternatively, an explanation of why a DMC is not needed	__p12_______
	21b	Description of any interim analyses and stopping guidelines, including who will have access to these interim results and make the final decision to terminate the trial	____n/a______
Harms	22	Plans for collecting, assessing, reporting, and managing solicited and spontaneously reported adverse events and other unintended effects of trial interventions or trial conduct	_____p10_______
Auditing	23	Frequency and procedures for auditing trial conduct, if any, and whether the process will be independent from investigators and the sponsor	______n/a______
**Ethics and dissemination**			
Research ethics approval	24	Plans for seeking research ethics committee/institutional review board (REC/IRB) approval	____p12________

Protocol amendments	25	Plans for communicating important protocol modifications (eg, changes to eligibility criteria, outcomes, analyses) to relevant parties (eg, investigators, REC/IRBs, trial participants, trial registries, journals, regulators)	_______n/a______
Consent or assent	26a	Who will obtain informed consent or assent from potential trial participants or authorised surrogates, and how (see Item 32)	_______p7______
	26b	Additional consent provisions for collection and use of participant data and biological specimens in ancillary studies, if applicable	______p10_______
Confidentiality	27	How personal information about potential and enrolled participants will be collected, shared, and maintained in order to protect confidentiality before, during, and after the trial	______p8______
Declaration of interests	28	Financial and other competing interests for principal investigators for the overall trial and each study site	______p14_______
Access to data	29	Statement of who will have access to the final trial dataset, and disclosure of contractual agreements that limit such access for investigators	_____n/a________
Ancillary and post-trial care	30	Provisions, if any, for ancillary and post-trial care, and for compensation to those who suffer harm from trial participation	______n/a_______
Dissemination policy	31a	Plans for investigators and sponsor to communicate trial results to participants, healthcare professionals, the public, and other relevant groups (eg, via publication, reporting in results databases, or other data sharing arrangements), including any publication restrictions	___p13_________
	31b	Authorship eligibility guidelines and any intended use of professional writers	_____n/a________
	31c	Plans, if any, for granting public access to the full protocol, participant-level dataset, and statistical code	______n/a______
**Appendices**			
Informed consent materials	32	Model consent form and other related documentation given to participants and authorised surrogates	______n/a______
Biological specimens	33	Plans for collection, laboratory evaluation, and storage of biological specimens for genetic or molecular analysis in the current trial and for future use in ancillary studies, if applicable	_______n/a______

For peer review only

*It is strongly recommended that this checklist be read in conjunction with the SPIRIT 2013 Explanation & Elaboration for important clarification on the items. Amendments to the protocol should be tracked and dated. The SPIRIT checklist is copyrighted by the SPIRIT Group under the Creative Commons "Attribution-NonCommercial-NoDerivs 3.0 Unported" license.

For peer review only

Figure 2. Schedule of enrolment, interventions, and assessments

	STUDY PERIOD					
	Enrolment	Allocation	Intervention period		Follow-up	Endpoint
**TIMEPOINT (weeks)**	*-1 week*	**0**	***1***	***20***	***21-25***	***26***
**ENROLMENT:**						
**Eligibility screen**	X					
**Informed school consent**	X					
**Randomisation**		X				
**INTERVENTIONS:**						
The CVI Project Intervention			◆————	————◆		
Control standard care			◆————	————◆		
**ASSESSMENTS:**						
Child PedsQL™ generic		X			X	
Child PedsQL™ Cognitive functioning		X			X	
School questionnaire		X			X	
Teacher self-efficacy		X			X	
Teacher Bartimeus CVI		X			X	
Teacher SDQ		X			X	
Teacher PedsQL™ cognitive functioning		X			X	
Teacher Dutton 5 Qs		X			X	
Parent SDQ		X			X	
Parent PedsQL™ Family Impact		X			X	
Parent health costs Qs		X			X	
Parent Dutton 5 Qs		X			X	
Classroom clutter		X			X	
SENCO interviews			X		X	
School staff interviews			X			
Parent interviews			X			
Eye Service staff interviews					X	
Document analysis		X			X	
Functional visual assessments				X		

For peer review only

For peer review only - http://bmjopen.bmj.com/site/about/guidelines.xhtml

BMJ Open

Improving outcomes for primary school children at risk of cerebral visual impairments (The CVI Project): Protocol of a feasibility study for a cluster-randomised controlled trial and health economic evaluation

Journal:	*BMJ Open*
Manuscript ID	bmjopen-2020-044830.R1
Article Type:	Protocol
Date Submitted by the Author:	20-Jan-2021

Complete List of Authors:	Williams, Cathy; University of Bristol, Bristol Medical School<br>Pease, Anna; University of Bristol, Bristol Medical School<br>Goodenough, Trudy; University of Bristol, Bristol Medical School<br>Breheny, Katie; University of Bristol, Bristol Medical School<br>Gaunt, Daisy; University of Bristol, Bristol Medical School<br>Sinai, Parisa; University of Bristol, Bristol Medical School<br>Watanabe, Rose; University of Bristol, Bristol Medical School

**Primary Subject Heading**:	Ophthalmology
Secondary Subject Heading:	Paediatrics, Ophthalmology, Epidemiology
Keywords:	Paediatric ophthalmology < OPHTHALMOLOGY, Developmental neurology & neurodisability < PAEDIATRICS, EPIDEMIOLOGY

SCHOLARONE™ Manuscripts

I, the Submitting Author has the right to grant and does grant on behalf of all authors of the Work (as defined in the below author licence), an exclusive licence and/or a non-exclusive licence for contributions from authors who are: i) UK Crown employees; ii) where BMJ has agreed a CC-BY licence shall apply, and/or iii) in accordance with the terms applicable for US Federal Government officers or employees acting as part of their official duties; on a worldwide, perpetual, irrevocable, royalty-free basis to BMJ Publishing Group Ltd ("BMJ") its licensees and where the relevant Journal is co-owned by BMJ to the co-owners of the Journal, to publish the Work in this journal and any other BMJ products and to exploit all rights, as set out in our licence.

The Submitting Author accepts and understands that any supply made under these terms is made by BMJ to the Submitting Author unless you are acting as an employee on behalf of your employer or a postgraduate student of an affiliated institution which is paying any applicable article publishing charge ("APC") for Open Access articles. Where the Submitting Author wishes to make the Work available on an Open Access basis (and intends to pay the relevant APC), the terms of reuse of such Open Access shall be governed by a Creative Commons licence – details of these licences and which Creative Commons licence will apply to this Work are set out in our licence referred to above.

Other than as permitted in any relevant BMJ Author's Self Archiving Policies, I confirm this Work has not been accepted for publication elsewhere, is not being considered for publication elsewhere and does not duplicate material already published. I confirm all authors consent to publication of this Work and authorise the granting of this licence.

For peer review only

Improving outcomes for primary school children at risk of cerebral visual impairments (The CVI Project): Protocol of a feasibility study for a cluster-randomised controlled trial and health economic evaluation

C Williams [1]; A S Pease[1]; T Goodenough [1]; K Breheny[3]; D Gaunt[2]; P Sinai[1] and R Watanabe[1]

[1] Centre for Academic Child Health, Bristol Medical School, University of Bristol, 1-5 Whiteladies Road, BS8 1NU
[2] Bristol Randomised Trials Collaboration, Population Health Sciences, Bristol Medical School, University of Bristol
[3] Population Health Sciences, Bristol Medical School, University of Bristol

Corresponding Author: C Williams, 1-5 Whiteladies Road, Bristol BS8 1NU
Cathy.Williams@bristol.ac.uk

C Williams (orcid.org/0000-0002-9133-2021)
A Pease
T Goodenough
R Watanabe
P Sinai
K Breheny

1-5 Whiteladies Road, Clifton, Bristol, UK, BS8 1NU

D Gaunt

Canynge Hall, 39 Whatley Road, Bristol, UK, BS8 2PS

Word count:3802

For peer review only - http://bmjopen.bmj.com/site/about/guidelines.xhtml

ABSTRACT

Introduction

Cerebral visual impairment (CVI) refers to a spectrum of brain-related vision problems. CVI is associated with poor educational and mental health outcomes. An intervention has been developed to help children with CVI, involving input from clinicians, teachers and parents. The effectiveness of this intervention needs to be evaluated. This study aims to guide any refinements to the intervention or the design of a future cluster-randomised trial that may be needed.

Methods and Analysis

This study will include all methods anticipated for a future cluster-randomised trial. Eight primary schools will be recruited and randomised to receive the intervention or carry on with usual practice. The intervention will comprise an information pack for schools and access to a local paediatric ophthalmology clinic (who are prepared to assess them for CVI), for up to 5% of participating children. Outcome assessments will be carried out at baseline (before randomisation) and after a 4 to 5 months intervention period. Assessments will include children's self-reported quality of life (QoL), their learning ability and behaviour as reported by teachers, and family functioning reported by parents. Cost data will include service use, family expenditure on additional support (e.g. private appointments, administration) and school spending and resource used in helping children with special educational needs or disability (SEND). A process evaluation (PE) will collect additional data relating to the implementation of the intervention and the trial processes, in the school and clinic settings. The protocol for the PE will be reported separately.

Ethics and Dissemination

Ethical permission was obtained from the University of Bristol Faculty of Health Sciences Ethical Committee. The results will inform the design of a future trial to assess the effectiveness and cost-effectiveness of the intervention and will be shared with participants, CVI-support groups and peer viewed journals.

Keywords: Cerebral Visual Impairment, Complex intervention, feasibility trial, process evaluation, child, Primary schools.

For peer review only

Strengths and Limitations of this study

All elements/methods proposed for the future cluster-randomised trial will be included, which will allow refinements to be made if needed

A detailed, mixed methods Process Evaluation (PE) is embedded

Children with CVI are not individually identified and the primary analysis will be instead a group presumed to be enriched with affected children, which will limit the statistical power

The duration of the study is limited to two terms so we cannot estimate longer-term changes in implementation or effect

There is no provision to interview General Practitioners (GPs) to understand their views on the suggested referral pathway

For peer review only

For peer review only - http://bmjopen.bmj.com/site/about/guidelines.xhtml

Introduction

Damage or poor function in the brain areas related to vision leads to brain-related visual impairment, also known as cerebral visual impairment or impairments (CVI) (1). There is not yet consensus on the specific type or severity of vision problems that should warrant a diagnosis of CVI, but there is general acceptance that brain-related vision disorders are important and a major cause of registrable blindness in the UK and other developed countries (2). A recent review suggested CVI was a verifiable visual impairment not attributable to ocular or optic nerve problems (3). Children with CVI may not be identified if they have good visual acuity (4) so are not detected in visual screening programmes recommended for Reception year (age 4-5) children. (5) They are therefore at risk of poor outcomes because of lack of support.

Simple strategies can be helpful for children with CVI, including de-cluttering presentations, using a window to isolate part of the page, or using plain backgrounds (6). Decluttering can also increase engagement with lessons for children without known problems (7). However, there are no recommendations for teachers regarding CVI or vision problems in general and if these have been diagnosed, the child is usually referred to a specialist teacher (a Qualified Teacher for the Visually Impaired, or QTVI).

In a recent study by our group, at least 3.4% of all the children in primary schools we surveyed had CVI-related vision problems (8). We found that none of the potential indicators of higher risk for CVI-related vision problems (red flags) we examined were very accurate: having extra educational help was 79% sensitive but only 48.9% specific, whilst by contrast a vision-specific questionnaire was 91% specific but had only 13% sensitivity.

Development of the complex intervention

We have developed a multi-level intervention that aims to improve outcomes for children with CVI. The intervention involves sharing information about the condition with the teachers, empowering them to try relevant strategies for children who are struggling and improving links between schools and the paediatric ophthalmology clinic.

Our previous study (8) has suggested that on average at least one child per class has CVI-related vision problems and that the questionnaires we used do not accurately identify affected children in a community setting. The intervention therefore emphasises to teachers that universal approaches (eg decluttering classrooms) will be helpful, as well as additional targeted approaches for children who are struggling. In this way all children are exposed to potentially helpful "vision-friendly" surroundings and there is less risk of a child getting no support because they are not yet diagnosed. A tiered approach using universal as well as targeted support has been recommended for other conditions affecting children, including anxiety and mental health problems. (9, 10)

Families have told us that referrals to the eye clinic and treatment there can be difficult if there is little understand of CVI. Another aspect of the intervention was therefore to provide an enhanced interface between the school and the eye clinic, by ensuring that the clinic was equipped and willing to accept referrals of children who may have CVI.

The school intervention pack materials were complied with advice from our professional advisory group (see below) and our local authority QTVIs and comprise:

For peer review only - http://bmjopen.bmj.com/site/about/guidelines.xhtml

(i) A short (30 min) presentation with audio commentary by the study PI (CW) giving an overview of CVI and how to help affected children and of the other materials provided, plus a transcript of the presentation.

(ii) Links to existing online resources about CVI

(iii) A "Toolkit"- a Word document detailing simple, inexpensive strategies (a) for universal use with whole class or school and (b) targeted measures to help individual children who are struggling with learning. These were reviewed and amended by our local QTVIs.

(iv) Some teaching resources (stories, suggested activities for children to do), matched to the curriculum, for teachers to use to help explain topics related to vision and light.

(vi) Guidance for teachers as to which children may have CVI and which may benefit from a referral to the eye clinic including suggested referral criteria.

(v) Letters for teachers to give to parents to show their GP, requesting referral to the local paediatric ophthalmology clinic because vision problems are suspected. At the request of the University Faculty Ethical committee, the number of study referrals was capped at 5% of participating children (to avoid over-burdening the GPs or the eye clinic) although GPs can make whatever referrals they want outside of the study, in the usual way.

The eye clinic intervention materials comprise:

(i) a validated questionnaire to score risk for CVI in at-risk children (11)

(ii) a protocol for vision tests to identify CVI-related vision problems, as used in our earlier study (8)

(iii) sample advice sheets on how to help children with CVI, adapted from existing websites and resources (12, 13) augmented with input from our local QTVIs

(iv) a link to a secure REDCap database (Research Electronic Data Capture, v9) to enter the results of the vision tests and print out a summary report to give the families, schools and for the clinic notes

As part of the process described by the Medical Research Council (MRC) for the development of complex interventions (14) we need to evaluate this intervention and see if in real life, the intervention need changing or adapting.

When and if we have evidence that the intervention is helpful, we will make the study materials including the advice sheets, available on request on our website (www.thecviproject.co.uk)

How the intervention might work

There are several pathways by which the intervention might potentially improve outcomes for children with CVI eg teachers adapting their teaching methods, or accurately identifying and referring affected children to the eye clinic. We have conceptualised these in a Logic model and

For peer review only - http://bmjopen.bmj.com/site/about/guidelines.xhtml

designed a Process Evaluation (PE) to collect data on what changes in school processes or attitudes if any, actually occur. We report the protocol for this separately (in press BMJ Open).

Aim and objectives of this feasibility cluster-randomised trial (cRCT)

The aim of this study is to assess the feasibility and acceptability of the new intervention and of the proposed trial methods, to inform the development of a future fully powered cluster-randomised trial.

Our specific objectives are to collect data on:

Intervention-related outcomes

1. How the intervention is implemented in practice - a detailed Process Evaluation (PE) has been designed to do this as reported in a separate protocol paper (ref)

2. Any changes in the outcome data (see Table 1) that may indicate an effect of the intervention, to help us calculate the sample size needed for the future trial

3. Attitudes of teachers and parents to the intervention and whether it is beneficial

Study design-related outcomes

We will document:

1. The response rate and yield from recruitment flyers, letters and emails sent, any reasons given for non-participation and retention of schools once recruited (once they have signed the study paperwork- see below)

2. The proportion and demographic predictors of parental non-agreement to data sharing in schools that have been recruited

3. The completeness of data from (a) child-report, (b) teacher-report and (c) parent questionnaires

4. The number of children suggested by the schools for a referral to the eye clinic, the proportion that attend the eye clinic and of those, the proportions who do and do not have any CVI-related vision problems

Methods and Analysis

Design

The design of this feasibility cRCT is shown in the flowchart in Figure 1. The processes for children referred to the eye clinic as part of the intervention are shown in Figure 2.

Figure 1: The CVI Project Feasibility Trial Flowchart

For peer review only - http://bmjopen.bmj.com/site/about/guidelines.xhtml

Figure 2: The process of referral to the eye clinic for intervention schools

Setting

The study will involve primary schools in three areas of England, UK: Gloucestershire, Somerset and Southampton. These areas were chosen because the local Paediatric Ophthalmologists have agreed to participate in the study. The study will be led by the study team in Bristol, UK.

Inclusion criteria

All mainstream state-funded Primary schools including academies, local authority maintained and free schools, within one hour of travel time to the local ophthalmology unit, in the three study areas will be eligible for inclusion.

Exclusion criteria

Schools who share a Special Educational Needs and Disability Co-ordinator (SENCo or SENDCo), where contamination between intervention to control schools might occur, unless they are part of a linked group of schools who all participate in the trial (and will be in the same arm).

Enrolment of schools

All schools in the study areas that meet the inclusion criteria will be sent an invitation letter and copies of the school and parent/carer information sheets. Invitation letters and information sheets will be sent out by post and email to Heads, SENCos and Heads of Governors. The letters and information sheets will explain the study and that schools will be reimbursed for the teachers' time completing outcome assessments.

Legal basis for conducting the study

The legal basis for the study will be the University of Bristol undertaking a Public task, as described in articles 6.1e and 9.2j of the EU-GDPR. It will not be on the basis of parental consent.

The Head Teacher will agree to participate on behalf of the children and staff in their school and will sign a Memorandum of Understanding (MOU) to this effect with clear descriptions of the expectations and obligations of the study team and the participating school. This will be non-binding and is written to foster mutual understanding of what is involved - there will be no steps taken if schools are unwilling or unable to carry out all their activities. A link to the full Privacy notice online will be provided to all parents and school staff and a summary of the Privacy notice is included in the Parent/ carer information sheet (PIS) and the School information sheet.

Any parent in a participating school will be able to withdraw their child's data from the study. This can be achieved by contacting the study team using details included in the PIS, or by completing a

For peer review only - http://bmjopen.bmj.com/site/about/guidelines.xhtml

Non-Agreement to Data Use form that they will all be sent with a PIS, plus a freepost envelope. They will be able to do this at any time up to the end of the study. In addition, if a child does not assent to filling in the child-report questionnaires, they do not have to. They will still be included in the study unless their parent has withdrawn them.

Study set-up and Baseline assessments

An Excel sheet will be completed by the school with name, gender, date of birth (dob), school pupil number (UPN), status regarding free school meals (FSM), siblings at the school and the level of and reason for extra educational help, for all children in Y3-5. Unique study identification numbers (IDs) for each child will be generated. The study team will then provide a Parent Information Sheet (PIS) and a questionnaire for each family, plus self-completion forms for the children, all with individual study ID numbers.

Outcome assessments were selected from the literature suggesting that unsupported CVI can impair children's learning (15, 16) and from a Core Outcome set (COS) study identifying outcomes relating to CVI in children that were rated as important by families and professionals (paper in preparation). The assessment tools to be used are summarised in Table 1**.** The children will be asked to complete the child-report PedsQL quality of life scales (17). The teachers will be asked to complete the PedsQL Cognitive scales which estimate the children's ability to learn (18) and Goodman's Strengths and Difficulties questionnaire(19). The parents will be asked to complete the PedsQL Family Impact Module (20) and a resource use questionnaire developed for the study (see below and a copy is given as Supplemental material). Both teachers and parents will also be asked to complete a short vision-specific questionnaire which elicits behaviours suggestive of CVI (21). Finally, the teachers will be asked to complete a questionnaire about their perception of their own teaching skills and abilities.

The study team will send or deliver to schools all the materials they need (school and parent information sheets, child questionnaires, parent questionnaires). The parent and child questionnaires will all have a front sheet giving the child's name, class and study ID number, whilst the actual questionnaires will have only the study ID. On completion, the front sheets will be removed and the data returned to the study team with study ID only, by freepost envelope for the parent questionnaires and child-report forms will be collected from the school by a member of the study team.

Teachers will get a link to an online questionnaire generated by a REDCap database and will complete one for each child in their class, plus answer the Self-efficacy questionnaire relating to their own teaching practice. A schedule showing when the assessments will be carried out is shown in Supplementary Materials.

Table 1. Assessment tools to be used

Respondent	Concept being investigated	Tool	Format

For peer review only - http://bmjopen.bmj.com/site/about/guidelines.xhtml

Child	Quality of Life	PedsQL Generic Core Scales:<br>*Child -report 8-12y*<br>*OR*<br>*Child report 5-7y (supported by adult report)*	Paper, in the classroom and supported by school staff<br><br>Which form the child completes is at discretion of their teacher
Teacher	Child learning ability	PedsQL Cognitive Scales	Online REDCap form– one per child
	Child behaviour at school	SDQ with impact supplement	Online REDCap form – one per child
	Child behaviour that may indicate CVIs	5-item CVI questionnaire	Online REDCap form– one per child
	Teacher perception of their own teaching	Self-Efficacy	Online REDCap form – one per teacher
Parents	Parental Quality of Life related to family	PedsQL Family Impact Module	Paper<br>Parents fill in one per child in the study
	Generic and child related costs and resource input	Family expenses questionnaire (designed for the study)	Paper<br>Parents fill in one per child in the study
	Child behaviour that may indicate CVIs	5-item CVI questionnaire	Paper<br>Parents fill in one per child in the study

Randomisation

After baseline data are collected, a statistician separate from the study will randomise the participating schools, with two stratification variables: (a) 15% or less of children having SEND vs >15% and (b) 3+ classes per year vs 2 or fewer classes per year.

The schools will be informed of their allocation by email and telephone call, all on the same day.

Post-randomisation

Study Schools

Schools allocated to the intervention group will be sent the CVI intervention pack (by post and by email) and the referral letters to use if they want to suggest a child is seen in the eye clinic. They will be asked to study these as soon as they can, however the actual timing and use of the packs will be examined in detail in the PE.

Control Schools

For peer review only - http://bmjopen.bmj.com/site/about/guidelines.xhtml

Schools allocated to the control group will be asked to carry on as normal

Duration of study period

For pragmatic reasons (limited availability of time and funds) the intervention period (the length of time between giving the schools the intervention pack and collecting the outcome data) will be two terms whereas in the full trial it is expected that the intervention period would be a full year.

Health Economic Evaluation

It is anticipated that the future trial will involve a cost-utility analysis (CUA), from a public sector perspective that includes family, education and health service costs. We will collect data from parents on their children's use of NHS primary and secondary care resource use and any additional costs incurred to support their child's health and educational needs. This will include time spent pursuing specialist appointments and other administrative tasks as well as costs related to their child's vision. A copy of this questionnaire is included as a Supplemental file.

We will collect data from schools about the resource they allocate to supporting children with SEND, in terms of numbers and hours of Learning Support Assistants (LSAs), costs of any special equipment, and requests for external specialists e.g. Speech and Language therapists. We will collect data from the eye clinics about numbers of study children who attend after referral by the teachers, the staff involved, the average time for appointments and the cost of equipment needed. The costs of delivering the intervention by schools will be explored in the process evaluation.

Blinding and contamination

It will not be possible to blind school staff as to which arm of the study they are in, or the fieldwork staff, however analysis of the quantitative data will be carried out by a separate statistician who is blind to group allocation. The primary outcome for the definitive trial is planned to be the child self-reported QoL which should be less subject to allocation bias than the teacher-report would be. The feasibility of collecting the child-report data is therefore an important outcome from this study. The intervention group schools will be asked not to share the intervention materials however it is recognised that contamination between arms may occur and the PE will investigate this. If there is sharing of information by schools or by parents, we will note this and decide what changes should be made to the parent information leaflets provided in the future trial.

Sample Size

As this is a feasibility cRCT, formal power calculations are not appropriate. Based on our previous study(8) , we aim to recruit enough primary schools that 1300 children are involved. Assuming data returns of 95% and a prevalence of 3.5% of CVI-related vision problems, this would give approximately 22 children with CVI-related vision problems each arm, most of whom are expected to be in the group of children having extra educational help. Depending on class sizes and structure, we estimate 8 schools will be recruited and randomised.

For peer review only - http://bmjopen.bmj.com/site/about/guidelines.xhtml

Analysis

Analyses will be mainly descriptive and will include 95% confidence intervals (95% CI) wherever appropriate. For between arm comparisons, an Intention-to-Treat approach will be used.

Recruitment

We will report the numbers of flyers and invitation letters sent by post and email, and the yield from these to see which produces the highest number of schools agreeing to take part. We will report the numbers and proportions of children whose parents elect not to share their data. We will compare these results with those published by other trials involving Primary schools and consider whether any changes in approach would be needed for a full trial.

Outcome assessment data

We will report the numbers and proportions of participating children for whom we receive child-report, teacher-report and parent-report outcome data at baseline and at follow-up. We will report the proportion of missing data within returned questionnaires.

We will examine and describe all the questionnaire data from children, teachers and parents. Although this is a feasibility study rather than the definitive trial, it will be useful to see the direction and magnitude of any change in the QoL or learning ability scores during the study. We do this for all children and for the sub-group having extra educational help, who we expect will have a higher prevalence of CVI-related vision problems based on our prevalence study in which 42% of children having extra help had one or more CVI-related vision problems (8). Therefore, we expect this group to be on average more sensitive to the effects of any vision-enhancing interventions they receive. As we cannot be sure who has CVI or CVI-related vision problems without examining all the participants, which would be unfeasible in real life, we will consider the group having extra educational help as a sample that is enriched with children who have CVI. We will also look specifically at the outcome data for any intervention group children referred to the eye clinic and diagnosed with CVI. We will estimate the number of children referred who do not have any CVI-related vision problems, as an indicator of how accurate the teacher referrals are.

We will use these data to guide the plans for the design and analysis of the future trial and estimate the numbers that will be needed for the trial to have appropriate statistical power.

Health Economic Evaluation

The health economic analysis will also be predominantly descriptive. Item-level missing data and the return of adult and child questionnaires will be explored. As the intervention is predominantly delivered outside of the NHS, data on individual level resource use in this study is mostly reliant upon parent report. The data quality will be explored to inform the refinement of questionnaires and utility of pursuing linked data collection in a future trial. Completion and return of school and NHS eye clinic data will also be examined.

There is extensive literature (22) mapping the PedsQL to the CHU9D, so we will estimate quality adjusted life years (QALYs) associated with any change in QoL. The possibility of alternative frameworks including broader outcomes, such as cost-consequence analyses (CCA), will also be explored.

For peer review only - http://bmjopen.bmj.com/site/about/guidelines.xhtml

Governance

The study will be managed day-to-day by the study team. Expert advice will be available from the study Advisory groups. Trial Steering Committee (TSC), chaired by an independent expert academic, will oversee the conduct of the study. The TSC will discuss the study results and findings with the study team and will advise on the final decision of whether to apply for funding for a full trial, with modifications to the intervention or study methods if indicated.

Patient and Public Involvement

Our Parents Advisory group (comprising parents of children with CVI) have advised on the design and materials for the study at several timepoints. At the outset they agreed that informing teachers about CVI and how to help affected children was an important intervention. They recommended including outcomes related to child learning ability as well as well-being. They recommended that the Control group of schools be offered the intervention materials at the end of the study and that the schools would need reimbursing for the teacher-time spent filling in questionnaires.

At subsequent meetings they revised the wording of the Parent information sheets. They commented on the assessment tools (Table 1) and recommended that parents might find some questions off-putting so we agreed that using just the SDQ Impact module was best for parents. Parents also suggested the term "vision -friendly" for measures like decluttering. They suggested emphasising to teachers the low cost and ease of the simple measures suggested and that they would be useful as evidence for Ofsted (the national schools' regulatory body), of interventions the teachers were using to support learners. They chose the Family Impact module above another questionnaire as a tool for capturing the family perspective.

We also have a Professionals Advisory group (a QTVI, a Community Paediatrician, an Educational Psychologist, a Head teacher, a SENCo, an Orthoptist and a Public Health consultant). This group have advised regularly on the study materials and design, specifically on the approach to schools, the scheduling of recruitment, the wording of the intervention pack materials and the referral process for children to get to the eye clinic.

Ethics and Dissemination

Ethical approval for the protocol of this study was obtained from the University of Bristol Faculty of Health Sciences Ethics Committee (FREC Ref: 89144) in August 2019. The Committee requested changes to the Protocol when first submitted: giving parents more detail about CVI in the PIS and capping the number of referrals to the eye clinic to prevent the GPs or eye clinic being overburdened. Once we had made these changes, a favourable opinion to the study was granted. The study is registered on the International Standard Randomised Controlled Trial Number registry (registration number: ISRCTN13762177). Approval from the Health Research Authority (HRA- Ref 19/HRA/6124) has been obtained.

Results from this study will be submitted to peer-reviewed journals and presented at scientific conferences. Summaries will be made available to participating schools, our advisory groups and family support group websites and social media platforms. A meeting for all participants and

For peer review only - http://bmjopen.bmj.com/site/about/guidelines.xhtml

collaborators will be held to present the findings. If indicated, the results will be used in a funding application for a future trial.

Trial status

Recruitment for this trial began in August 2019 and closed in December 2019. Baseline data were collected in December 2019 and January 2020, then the schools were randomised and informed of their group allocation. Intervention packs were delivered to schools in March 2020. The study was paused in March 2020 because of the corona virus pandemic (covid19). At the time of submission (14 September 2020), we have obtained Faculty Ethical committee (FREC) and Health Research Authority (HRA) permission to restart the study, with minor amendments to the protocol in line with covid19 precautions. Follow-up data will be collected in January 2021. The covid19 amendments will be reported with the results.

Acknowledgements

We are very grateful to Mai Baquedano for her help and innovation with the REDCap database, to Cassandra Wye for the stories and learning resources and to Professor Innes Cuthill for advice and support with measuring visual clutter.

Author contributions

CW designed and led the study, with input from all authors. AP and TG led and designed the Process Evaluation. PS provided governance support and RW managed the databases. KB designed and led the Economic Evaluation and DG provided statistical support. All authors contributed to and approved the final manuscript.

Funding

This study presents independent research funded by the National Institute for Health Research (NIHR) Senior Fellowship award SRF_2015_08_005.

Disclaimer

The views expressed are those of the author(s) and not necessarily those of the NHS, the NIHR or the Department of Health and Social Care.

For peer review only - http://bmjopen.bmj.com/site/about/guidelines.xhtml

Competing Interests

None

For peer review only

For peer review only - http://bmjopen.bmj.com/site/about/guidelines.xhtml

References

1. Dutton GN. Cognitive vision, its disorders and differential diagnosis in adults and children: knowing where and what things are. Eye (London, England). 2003;17(3):289-304.
2. Rahi JS, Cable N. Severe visual impairment and blindness in children in the UK. The Lancet. 2003;362(9393):1359-65.
3. Sakki HEA, Dale NJ, Sargent J, Perez-Roche T, Bowman R. Is there consensus in defining childhood cerebral visual impairment? A systematic review of terminology and definitions. The British journal of ophthalmology. 2018;102(4):424-32.
4. van Genderen M, Dekker M, Pilon F, Bals I. Diagnosing cerebral visual impairment in children with good visual acuity. Strabismus. 2012;20(2):78-83.
5. Solebo AL, Cumberland PM, Rahi JS. Whole-population vision screening in children aged 4-5 years to detect amblyopia. Lancet. 2015;385(9984):2308-19.
6. Philip SS, Dutton GN. Identifying and characterising cerebral visual impairment in children: a review. Clinical & experimental optometry. 2014;97(3):196-208.
7. Fisher AV, Godwin KE, Seltman H. Visual environment, attention allocation, and learning in young children: when too much of a good thing may be bad. Psychol Sci. 2014;25(7):1362-70.
8. Williams C, Pease A, Warnes P, Harrison S, Pilon F, Hyvarinen J, et al. Cerebral visual impairment (CVI)-related vision problems in children attending primary schools in England: Cross-sectional survey with nested case-finding study. Developmental Medicine & Child Neurology. 2020;in press.
9. Dodge KA. Annual Research Review: Universal and targeted strategies for assigning interventions to achieve population impact. J Child Psychol Psychiatry. 2020;61(3):255-67.
10. Etherington C, Costello S. Comparing Universal and Targeted Delivery of a Mindfulness-Based Program for Anxiety in Children. Journal of Psychologists and Counsellors in Schools. 2019;29:22-38.
11. Ortibus E, Laenen A, Verhoeven J, De Cock P, Casteels I, Schoolmeesters B, et al. Screening for cerebral visual impairment: value of a CVI questionnaire. Neuropediatrics. 2011;42(4):138-47.
12. The TeachCVI Partnership [Available from: https://www.teachcvi.net/.
13. University of Ulster. CVI Resources [Available from: https://www.ulster.ac.uk/research/topic/biomedical-sciences/research/optometry-and-vision-science-research-group/vision-resources/professionals/cerebral-visual-impairment-assessment.
14. Craig P, Dieppe P, Macintyre S, Michie S, Nazareth I, Petticrew M, et al. Developing and evaluating complex interventions: the new Medical Research Council guidance. BMJ. 2008;337:a1655.
15. Molloy CS, Di Battista AM, Anderson VA, Burnett A, Lee KJ, Roberts G, et al. The contribution of visual processing to academic achievement in adolescents born extremely preterm or extremely low birth weight. Child Neuropsychol. 2017;23(3):361-79.
16. Williams C, Northstone K, Sabates R, Feinstein L, Emond A, Dutton GN. Visual perceptual difficulties and under-achievement at school in a large community-based sample of children. PLoS One. 2011;6(3):e14772.
17. Varni JW, Seid M, Kurtin PS. PedsQL 4.0: reliability and validity of the Pediatric Quality of Life Inventory version 4.0 generic core scales in healthy and patient populations. Med Care. 2001;39(8):800-12.
18. Varni JW, Limbers CA, Sorensen LG, Neighbors K, Martz K, Bucuvalas JC, et al. PedsQL Cognitive Functioning Scale in pediatric liver transplant recipients: feasibility, reliability, and validity. Qual Life Res. 2011;20(6):913-21.
19. Goodman R. The extended version of the Strengths and Difficulties Questionnaire as a guide to child psychiatric caseness and consequent burden. J Child Psychol Psychiatry. 1999;40(5):791-9.

For peer review only - http://bmjopen.bmj.com/site/about/guidelines.xhtml

20. Varni JW, Sherman SA, Burwinkle TM, Dickinson PE, Dixon P. The PedsQL Family Impact Module: preliminary reliability and validity. Health Qual Life Outcomes. 2004;2:55.
21. Gorrie F, Goodall K, Rush R, Ravenscroft J. Towards population screening for Cerebral Visual Impairment: Validity of the Five Questions and the CVI Questionnaire. PLoS One. 2019;14(3):e0214290.
22. Sweeney R, Chen G, Gold L, Mensah F, Wake M. Mapping PedsQL(TM) scores onto CHU9D utility scores: estimation, validation and a comparison of alternative instrument versions. Qual Life Res. 2020;29(3):639-52.

For peer review only

For peer review only - http://bmjopen.bmj.com/site/about/guidelines.xhtml

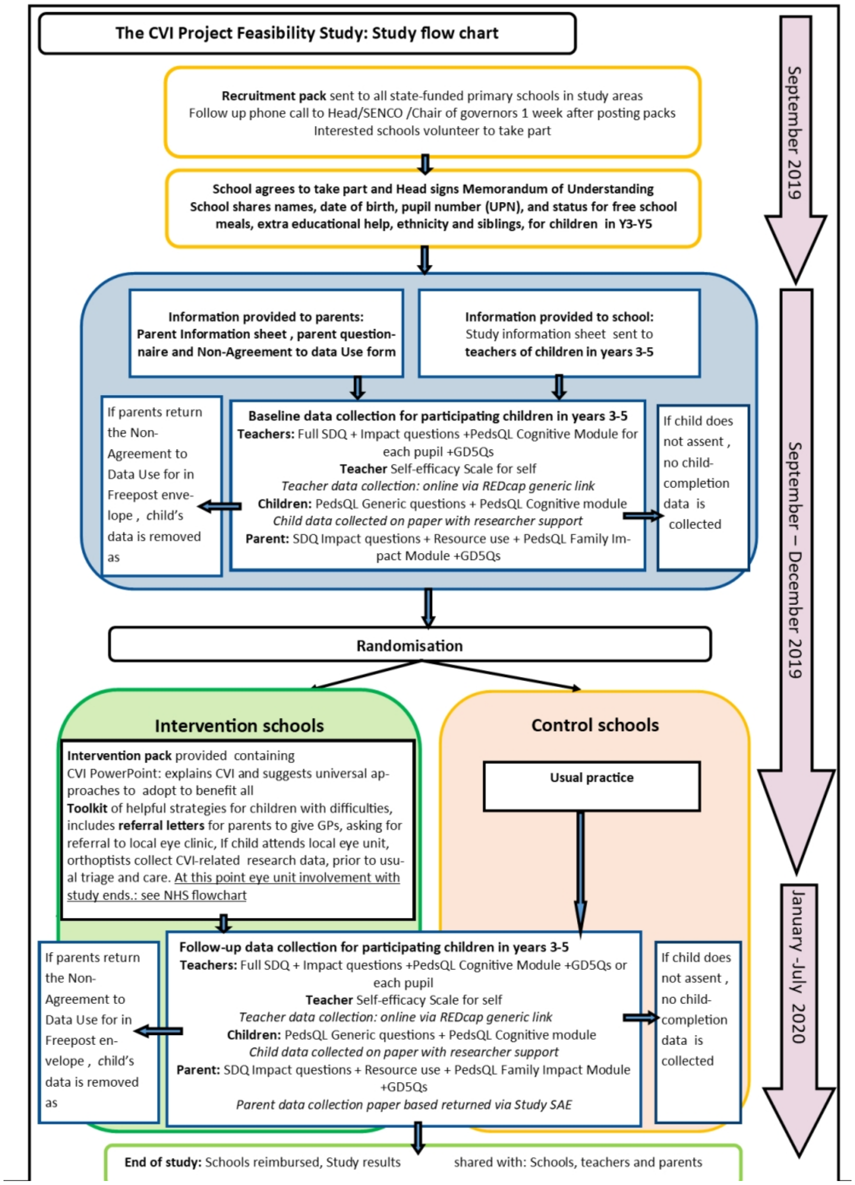
The CVI Project Feasibility Study: Study flow chart
September 2019
Recruitment pack sent to all state-funded primary schools in study areas
Follow up phone call to Head/SENCO /Chair of governors 1 week after posting packs
Interested schools volunteer to take part
School agrees to take part and Head signs Memorandum of Understanding
School shares names, date of birth, pupil number (UPN), and status for free school meals, extra educational help, ethnicity and siblings, for children in Y3-Y5
September – December 2019
Information provided to parents: Parent Information sheet , parent questionnaire and Non-Agreement to data Use form
Information provided to school: Study information sheet sent to teachers of children in years 3-5
If parents return the Non-Agreement to Data Use for in Freepost envelope , child's data is removed as
Baseline data collection for participating children in years 3-5
Teachers: Full SDQ + Impact questions +PedsQL Cognitive Module for each pupil +GD5Qs
Teacher Self-efficacy Scale for self
Teacher data collection: online via REDcap generic link
Children: PedsQL Generic questions + PedsQL Cognitive module
Child data collected on paper with researcher support
Parent: SDQ Impact questions + Resource use + PedsQL Family Impact Module +GD5Qs
If child does not assent , no child-completion data is collected
Randomisation
Intervention schools
Intervention pack provided containing
CVI PowerPoint: explains CVI and suggests universal approaches to adopt to benefit all
Toolkit of helpful strategies for children with difficulties, includes referral letters for parents to give GPs, asking for referral to local eye clinic, If child attends local eye unit, orthoptists collect CVI-related research data, prior to usual triage and care. At this point eye unit involvement with study ends.: see NHS flowchart
Control schools
Usual practice
January -July 2020
If parents return the Non-Agreement to Data Use for in Freepost envelope , child's data is removed as
Follow-up data collection for participating children in years 3-5
Teachers: Full SDQ + Impact questions +PedsQL Cognitive Module +GD5Qs or each pupil
Teacher Self-efficacy Scale for self
Teacher data collection: online via REDcap generic link
Children: PedsQL Generic questions + PedsQL Cognitive module
Child data collected on paper with researcher support
Parent: SDQ Impact questions + Resource use + PedsQL Family Impact Module +GD5Qs
Parent data collection paper based returned via Study SAE
If child does not assent , no child-completion data is collected
End of study: Schools reimbursed, Study results shared with: Schools, teachers and parents

Figure 1

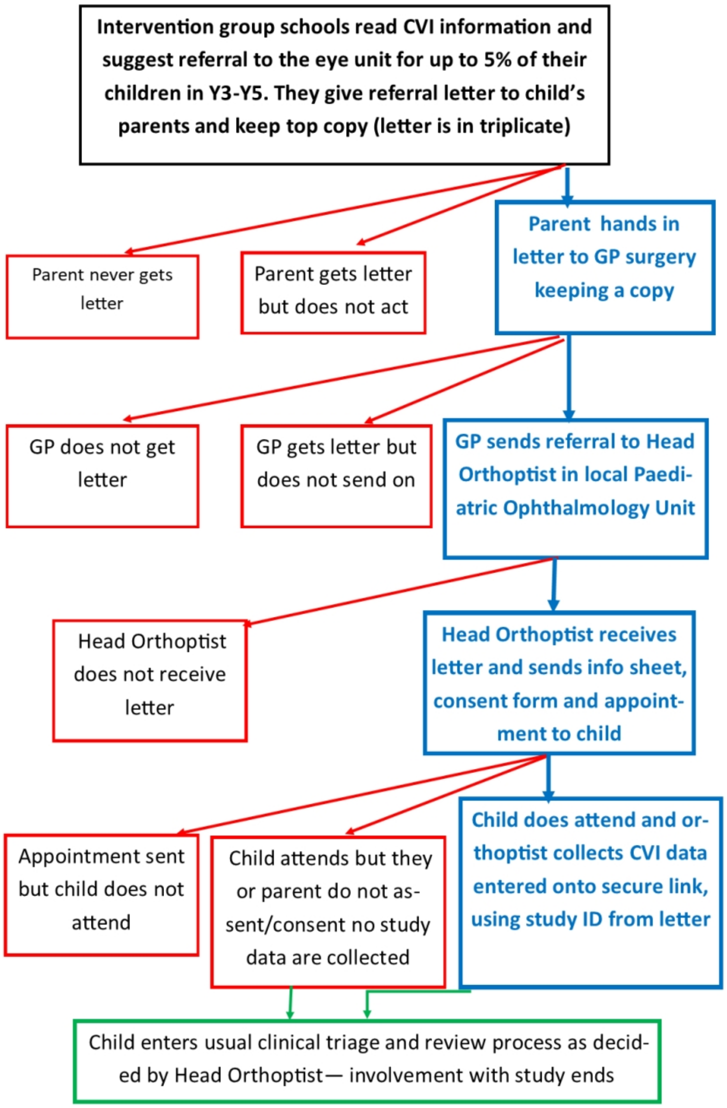
Intervention group schools read CVI information and suggest referral to the eye unit for up to 5% of their children in Y3-Y5. They give referral letter to child's parents and keep top copy (letter is in triplicate)
Parent never gets letter
Parent gets letter but does not act
Parent hands in letter to GP surgery keeping a copy
GP does not get letter
GP gets letter but does not send on
GP sends referral to Head Orthoptist in local Paediatric Ophthalmology Unit
Head Orthoptist does not receive letter
Head Orthoptist receives letter and sends info sheet, consent form and appointment to child
Appointment sent but child does not attend
Child attends but they or parent do not assent/consent no study data are collected
Child does attend and orthoptist collects CVI data entered onto secure link, using study ID from letter
Child enters usual clinical triage and review process as decided by Head Orthoptist— involvement with study ends

Figure 2

CVI Project Parent/Guardian Resource Use Questionnaire

Thank you very much for taking the time to complete this CVI Project parent/guardian resource use questionnaire.

Your answers will be treated in the strictest confidence and will not be shared with anyone outside of the research team. We appreciate you taking part.

We are interested in finding out which school, health and social care services your child has had access to in the last 12 months

1. Please indicate below how many times (if at all) your child has used, or been involved with, any of the services listed below in the last 12 months.
   If your child has not been in contact with a particular service, please enter '0' rather than leave it blank.

**Service**	**Total number of contacts**
Hospital outpatient visit (**vision related**, for example, ophthalmologist, orthoptist, hospital optician)	
High street optician	
General Practitioner (GP) (**vision related**)	
QTVI (Qualified Teacher of the Visually Impaired)	

**Service**	**Total number of contacts**
Hospital outpatient visit (**not vision related**)	
General Practitioner (GP) (**not vision related**)	
Paediatrician	
Health visitor	
Accident and Emergency (A&E) Visit	
Social Worker	
Hospital stay	
For hospital stay, please record the total number of nights your child has spent in hospital over the last 12 months	Number of nights:
Speech and language therapist	
Hearing specialist	
Occupational therapist	
Physiotherapist	
Child and Adolescent Mental Health Service (CAMHS)	
Educational psychologist	
Dentist	
Police	

For peer review only - http://bmjopen.bmj.com/site/about/guidelines.xhtml

If you run out of space, please use the following page to provide additional information.

2. Has your child used any other health and social care services that are not listed in the table above? If so please let us know:

Other service 1: ____

No. of contacts: __________

Other service 2: ____

No. of contacts: __________

3. In the <u>last 12 months</u>, have you **paid out of your own pocket** (not provided free) for any **<u>services</u>** related to those in the tables or listed above (e.g. private outpatient visit, counselling, homeopathy, educational psychologist)?

Service 1: ____

Total spend: __________

Service 2: ____

Total spend: __________

4. In the <u>last 12 months</u>, have you **paid out of your own pocket** (not provided free) for any **<u>equipment</u>** related to those in the table or listed above (e.g. additional costs for glasses)?

Item 1: ____

Total spend: __________

Item 2: ____

Total spend: __________

5. In the <u>last 12 months</u>, did you have to take time off work or your usual daily activities due to your child being off school? (For example, time off due to child's illness, behavioural problems, attending appointments etc.)

Person	NO	YES	If YES, how many days
**Yourself**			
**Partner**			
**Other please say who:**			

Thank you very much for your time.

FUNDED BY NIHR | National Institute for Health Research

University of BRISTOL

THE CVI PROJECT

For peer review only

For peer review only - http://bmjopen.bmj.com/site/about/guidelines.xhtml

Schedule of enrolment, interventions, and assessments

	STUDY PERIOD					
	Enrolment	Allocation	Intervention period		Follow-up	Endpoint
**TIMEPOINT (weeks)**	***-1 week***	**0**	***1***	***20***	***21-25***	***26***
**ENROLMENT:**						
**Eligibility screen**	X					
**Informed school consent**	X					
**Randomisation**		X				
**INTERVENTIONS:**						
The CVI Project Intervention						
Control standard care						
**ASSESSMENTS:**						
Child PedsQL™ generic		X			X	
Child PedsQL™ Cognitive functioning		X			X	
School questionnaire		X			X	
Teacher self-efficacy		X			X	
Teacher Bartimeus CVI		X			X	
Teacher SDQ		X			X	
Teacher PedsQL™ cognitive functioning		X			X	
Teacher Dutton 5 Qs		X			X	
Parent SDQ		X			X	
Parent PedsQL™ Family Impact		X			X	
Parent health costs Qs		X			X	
Parent Dutton 5 Qs		X			X	
Classroom clutter		X			X	
SENCO interviews			X		X	
School staff interviews			X			
Parent interviews			X			
Eye Service staff interviews					X	
Document analysis		X			X	
Functional visual assessments				X		

For peer review only

SPIRIT

STANDARD PROTOCOL ITEMS: RECOMMENDATIONS FOR INTERVENTIONAL TRIALS

SPIRIT 2013 Checklist: Recommended items to address in a clinical trial protocol and related documents*

Section/item	Item No	Description	Addressed on page number
**Administrative information**			
Title	1	Descriptive title identifying the study design, population, interventions, and, if applicable, trial acronym	Title___________
Trial registration	2a	Trial identifier and registry name. If not yet registered, name of intended registry	_p13___________
	2b	All items from the World Health Organization Trial Registration Data Set	___________
Protocol version	3	Date and version identifier	__p13__________
Funding	4	Sources and types of financial, material, and other support	__p14___________
Roles and responsibilities	5a	Names, affiliations, and roles of protocol contributors	__p1 and p13____
	5b	Name and contact information for the trial sponsor	______________
	5c	Role of study sponsor and funders, if any, in study design; collection, management, analysis, and interpretation of data; writing of the report; and the decision to submit the report for publication, including whether they will have ultimate authority over any of these activities	______________
	5d	Composition, roles, and responsibilities of the coordinating centre, steering committee, endpoint adjudication committee, data management team, and other individuals or groups overseeing the trial, if applicable (see Item 21a for data monitoring committee)	______________

For peer review only

Introduction

Background and rationale	6a	Description of research question and justification for undertaking the trial, including summary of relevant studies (published and unpublished) examining benefits and harms for each intervention	____p4_______
	6b	Explanation for choice of comparators	____p12________
Objectives	7	Specific objectives or hypotheses	____p6_____
Trial design	8	Description of trial design including type of trial (eg, parallel group, crossover, factorial, single group), allocation ratio, and framework (eg, superiority, equivalence, noninferiority, exploratory)	____pp6-10__

Methods: Participants, interventions, and outcomes

Study setting	9	Description of study settings (eg, community clinic, academic hospital) and list of countries where data will be collected. Reference to where list of study sites can be obtained	____p6______
Eligibility criteria	10	Inclusion and exclusion criteria for participants. If applicable, eligibility criteria for study centres and individuals who will perform the interventions (eg, surgeons, psychotherapists)	_____pp6=7_____
Interventions	11a	Interventions for each group with sufficient detail to allow replication, including how and when they will be administered	____p8_________
	11b	Criteria for discontinuing or modifying allocated interventions for a given trial participant (eg, drug dose change in response to harms, participant request, or improving/worsening disease)	_____n/a_______
	11c	Strategies to improve adherence to intervention protocols, and any procedures for monitoring adherence (eg, drug tablet return, laboratory tests)	____p10________
	11d	Relevant concomitant care and interventions that are permitted or prohibited during the trial	____n/a________
Outcomes	12	Primary, secondary, and other outcomes, including the specific measurement variable (eg, systolic blood pressure), analysis metric (eg, change from baseline, final value, time to event), method of aggregation (eg, median, proportion), and time point for each outcome. Explanation of the clinical relevance of chosen efficacy and harm outcomes is strongly recommended	___p8______
Participant timeline	13	Time schedule of enrolment, interventions (including any run-ins and washouts), assessments, and visits for participants. A schematic diagram is highly recommended (see Figure)	see Figure________

For peer review only - http://bmjopen.bmj.com/site/about/guidelines.xhtml

Sample size	14	Estimated number of participants needed to achieve study objectives and how it was determined, including clinical and statistical assumptions supporting any sample size calculations	_p11___________
Recruitment	15	Strategies for achieving adequate participant enrolment to reach target sample size	_p7___________

Methods: Assignment of interventions (for controlled trials)

Allocation:

Sequence generation	16a	Method of generating the allocation sequence (eg, computer-generated random numbers), and list of any factors for stratification. To reduce predictability of a random sequence, details of any planned restriction (eg, blocking) should be provided in a separate document that is unavailable to those who enrol participants or assign interventions	__p9__________
Allocation concealment mechanism	16b	Mechanism of implementing the allocation sequence (eg, central telephone; sequentially numbered, opaque, sealed envelopes), describing any steps to conceal the sequence until interventions are assigned	_p9____________
Implementation	16c	Who will generate the allocation sequence, who will enrol participants, and who will assign participants to interventions	_p9____________
Blinding (masking)	17a	Who will be blinded after assignment to interventions (eg, trial participants, care providers, outcome assessors, data analysts), and how	_p11___________
	17b	If blinded, circumstances under which unblinding is permissible, and procedure for revealing a participant's allocated intervention during the trial	__n/a__________

Methods: Data collection, management, and analysis

Data collection methods	18a	Plans for assessment and collection of outcome, baseline, and other trial data, including any related processes to promote data quality (eg, duplicate measurements, training of assessors) and a description of study instruments (eg, questionnaires, laboratory tests) along with their reliability and validity, if known. Reference to where data collection forms can be found, if not in the protocol	P8_________

	18b	Plans to promote participant retention and complete follow-up, including list of any outcome data to be collected for participants who discontinue or deviate from intervention protocols	_____p10_______
Data management	19	Plans for data entry, coding, security, and storage, including any related processes to promote data quality (eg, double data entry; range checks for data values). Reference to where details of data management procedures can be found, if not in the protocol	____p8________
Statistical methods	20a	Statistical methods for analysing primary and secondary outcomes. Reference to where other details of the statistical analysis plan can be found, if not in the protocol	_p11-12_______
	20b	Methods for any additional analyses (eg, subgroup and adjusted analyses)	p11-12________
	20c	Definition of analysis population relating to protocol non-adherence (eg, as randomised analysis), and any statistical methods to handle missing data (eg, multiple imputation)	_n/a_________
**Methods: Monitoring**			
Data monitoring	21a	Composition of data monitoring committee (DMC); summary of its role and reporting structure; statement of whether it is independent from the sponsor and competing interests; and reference to where further details about its charter can be found, if not in the protocol. Alternatively, an explanation of why a DMC is not needed	__p12_______
	21b	Description of any interim analyses and stopping guidelines, including who will have access to these interim results and make the final decision to terminate the trial	____n/a______
Harms	22	Plans for collecting, assessing, reporting, and managing solicited and spontaneously reported adverse events and other unintended effects of trial interventions or trial conduct	_____p10_______
Auditing	23	Frequency and procedures for auditing trial conduct, if any, and whether the process will be independent from investigators and the sponsor	______n/a______
**Ethics and dissemination**			
Research ethics approval	24	Plans for seeking research ethics committee/institutional review board (REC/IRB) approval	____p12________

Protocol amendments	25	Plans for communicating important protocol modifications (eg, changes to eligibility criteria, outcomes, analyses) to relevant parties (eg, investigators, REC/IRBs, trial participants, trial registries, journals, regulators)	_______n/a______
Consent or assent	26a	Who will obtain informed consent or assent from potential trial participants or authorised surrogates, and how (see Item 32)	_______p7______
	26b	Additional consent provisions for collection and use of participant data and biological specimens in ancillary studies, if applicable	______p10_______
Confidentiality	27	How personal information about potential and enrolled participants will be collected, shared, and maintained in order to protect confidentiality before, during, and after the trial	______p8______
Declaration of interests	28	Financial and other competing interests for principal investigators for the overall trial and each study site	______p14_______
Access to data	29	Statement of who will have access to the final trial dataset, and disclosure of contractual agreements that limit such access for investigators	_____n/a________
Ancillary and post-trial care	30	Provisions, if any, for ancillary and post-trial care, and for compensation to those who suffer harm from trial participation	______n/a_______
Dissemination policy	31a	Plans for investigators and sponsor to communicate trial results to participants, healthcare professionals, the public, and other relevant groups (eg, via publication, reporting in results databases, or other data sharing arrangements), including any publication restrictions	___p13__________
	31b	Authorship eligibility guidelines and any intended use of professional writers	_____n/a________
	31c	Plans, if any, for granting public access to the full protocol, participant-level dataset, and statistical code	______n/a______
**Appendices**			
Informed consent materials	32	Model consent form and other related documentation given to participants and authorised surrogates	______n/a______
Biological specimens	33	Plans for collection, laboratory evaluation, and storage of biological specimens for genetic or molecular analysis in the current trial and for future use in ancillary studies, if applicable	_______n/a______

For peer review only - http://bmjopen.bmj.com/site/about/guidelines.xhtml

*It is strongly recommended that this checklist be read in conjunction with the SPIRIT 2013 Explanation & Elaboration for important clarification on the items. Amendments to the protocol should be tracked and dated. The SPIRIT checklist is copyrighted by the SPIRIT Group under the Creative Commons "Attribution-NonCommercial-NoDerivs 3.0 Unported" license.

For peer review only

For peer review only - http://bmjopen.bmj.com/site/about/guidelines.xhtml
